# Correction: Public attitudes to potential synthetic cells applications: Pragmatic support and ethical acceptance

**DOI:** 10.1371/journal.pone.0350696

**Published:** 2026-05-29

**Authors:** The PLOS One Staff

## Notice of Republication

This article was republished on April 21 2026, to correct errors in the references. In the list of references, numbers 12–29 appeared in the wrong order. Please download this article again to view the correct version. The originally published, uncorrected article and the republished, corrected articles are provided here for reference.

## Supporting information

S1 File Originally published, uncorrected article. (PDF)

S2 File Republished, corrected article. (PDF)
