# Supplementary material for: Public attitudes to potential synthetic cells applications: Pragmatic support and ethical acceptance
Source: PLoS One. 2025 Feb 27;20(2):e0319337. doi: 10.1371/journal.pone.0319337 (PMC11867391; doi:10.1371/journal.pone.0319337)

Public Attitudes to Potential Synthetic Cells Applications: Pragmatic Support and Ethical Acceptance

O. Rook[1, 2], H. Zwart[2], M. Dogterom[1]

[1]Department of Bionanoscience, Kavli Institute of Nanoscience, Delft University of Technology, Delft, the Netherlands. [2]Erasmus School of Philosophy, Erasmus University Rotterdam, Rotterdam, the Netherlands.

Email: o.rook@tudelft.nl

Supplementary Information

Table S1
Demographics/Population characteristics.

Abbreviations here and below: Czech Republic (CZ), Germany (DE), Spain (ES), France (FR), Greece (GR), Hungary (HU), Italy (IT), Netherlands (NL), Poland (PL), Romania (RO), Sweden (SE), Turkey (TR), United Kingdom (UK).

	CZ		DE		ES		FR		GR		HU	
**Gender**												
Female	330	51%	331	51%	319	52%	337	50%	315	53%	318	53%
Male	318	49%	319	49%	296	48%	336	50%	285	48%	282	47%
Other	0	0%	1	0%	0	0%	0	0%	0	0%	0	0%
n.a.	0	0%	0	0%	1	0%	0	0%	0	0%	0	0%
**Age**												
18-24	52	8%	67	10%	55	9%	68	10%	54	9%	54	9%
25-34	98	15%	99	15%	80	13%	101	15%	77	13%	94	16%
35-44	122	19%	103	16%	109	18%	108	16%	103	17%	108	18%
45-54	116	18%	107	16%	126	20%	115	17%	109	18%	111	19%
55-64	98	15%	118	18%	100	16%	107	16%	95	16%	89	15%
65+	162	25%	157	24%	146	24%	174	26%	162	27%	144	24%
**Education**												
Low	78	12%	130	20%	302	49%	175	26%	179	30%	126	21%
Middle	424	65%	325	50%	142	23%	296	44%	260	43%	342	57%
High	146	23%	196	30%	172	28%	202	30%	161	27%	132	22%
**Total**	648	100%	651	100%	616	100%	673	100%	600	100%	600	100%

	IT		NL		PL		RO		SE		TR		UK	
**Gender**														
Female	319	52%	320	51%	388	52%	339	51%	345	52%	352	50%	289	49%
Male	296	48%	310	49%	359	48%	323	49%	322	48%	345	49%	290	49%
Other	2	0%	1	0%	1	0%	2	0%	0	0%	2	0%	7	1%
n.a.	0	0%	1	0%	1	0%	1	0%	1	0%	1	0%	4	1%
**Age**														
18-24	49	8%	71	11%	68	9%	73	11%	63	9%	69	10%	90	15%
25-34	83	13%	103	16%	128	17%	114	17%	101	15%	125	18%	122	21%
35-44	92	15%	93	15%	148	20%	103	15%	120	18%	113	16%	125	21%
45-54	114	18%	106	17%	118	16%	122	18%	134	20%	108	15%	100	17%
55-64	106	17%	107	17%	119	16%	102	15%	92	14%	106	15%	77	13%
65+	173	28%	152	24%	168	22%	151	23%	158	24%	179	26%	76	13%
**Education**														
Low	284	46%	185	29%	112	15%	153	23%	193	29%	189	27%	325	55%
Middle	247	40%	252	40%	457	61%	279	42%	373	56%	301	43%	116	20%
High	86	14%	195	31%	180	24%	233	35%	102	15%	210	30%	149	25%
**Total**	617	100%	632	100%	749	100%	665	100%	668	100%	700	100%	590	100%

Table S2
Criteria for excluding survey data from statistical and/or textual analysis.

Textual response	Example(s)	Likert scale responses	Textual analysis	Survey ID (Anonymized record no., per country code)	Total No.
Missing (it was not compulsory to enter text)		include	exclude	--	--
Unqualified statement suggesting one has nothing to say/add	that's it; that's all; pass; hmm; no; not; none	include	include	--	--
Unqualified statement to avoid answer	hi; hallo; because; yes; just; ok; simply; so; therefore; very well	include	exclude	CZ-026, CZ-037, CZ-213, CZ-284, DE-448, DE-487, DE-575, DE-580, DE-587, DE-598, FR-514, FR-658, ES-173, GR-211, GR-213, GR-457, GR-493, GR-543, GR-551, GR-561, GR-563, GR-591, HU-015, HU-092, HU-195, HU-358, HU-429, HU-510, HU-512, HU-526, HU-547, HU-555, HU-560, IT-073, IT-252, IT-284, NL-249, PL-032, PL-050, PL-179, PL-233, PL-358, PL-368, PL-650, RO-026, RO-227, RO-262, RO-282, RO-320, RO-334, RO-527, RO-551, RO-615, UK-067, UK-139	55
Gibberish	Uhvvfgbbgg; om	exclude	exclude	CZ-321, CZ-374, ES-480, IT-286, IT-424, RO-138, RO-571, RO-647, SE-156, UK-320	10
Incomplete/faulted sentence where the statement still makes sense	"The fear is that instead of doing good in the end [...] for all humanity"	include	include	--	--
Incomplete/faulted sentence where the statement does not make sense	"think it's doramyl"	include	exclude	ES-210, ES-280, PL-074, TR-409	4
Nonsensical text (e.g., a copy/pasted advertisement line)	"Super price is quoted for a few days a week"	exclude	exclude	CZ-452, CZ-498, PL-423, PL-467, TR-104, TR-224, TR-374	7
Coherent text not related to the topic	"Because you can contact the other person from all over the world"; "Because I'm a narcissistic person"	exclude	exclude	PL-026, PL-078, PL-296, PL-351, PL-703, RO-467, UK-260	7
statement expressing no interest/involvement in the topic	I don't care	include	include	--	--
statement expressing cynical attitude and/or no interest in the research	I don't care a damn;<br>I don't know Hitler;<br>I don't now, but I love you and I want moneyy;<br>my love	exclude	exclude	CZ-309, CZ-588, PL-393, PL-465, RO-588	5
vulgar language expressing one's attitude	it's all bullshit	include	include	--	--
respondent got distracted and forgot what it was about	"my child was babbling through it and forgot what it was about"	exclude	exclude	NL-084	1
Incomplete response erroneously saved by Qualtrics		exclude	exclude	TR-434	1
TOTAL EXCLUDED		31	90		90

Table S3

Sample size per country before and after removing invalid entries.

	Entries	After cleaning
**CZ**	648	642
**DE**	651	651
**ES**	616	615
**FR**	673	673
**GR**	600	600
**HU**	600	600
**IT**	617	615
**NL**	632	631
**PL**	749	740
**RO**	668	663
**SE**	700	699
**TR**	594	590
**UK**	665	663
**Total**	8413	8382

Table S4

Mean acceptability of SC-based applications and perceived willingness to use them: Anticancer therapy (V1), Conversion of CO_2 emissions to biofuel (V2) and Industrial waste recycling (V3).

The table shows Likert scale scores as numeric values: -2 for extremely unacceptable (Q1) or unlikely (Q2), -1 for somewhat unacceptable/unlikely, 0 for neither unacceptable/unlikely, nor acceptable/likely, 1 for somewhat acceptable/likely, and 2 for extremely acceptable/likely.

Q1: Acceptability.

	N	Mean	Std. Deviation
1 V1	2851	1.25	.992
2 V2	2759	.81	1.071
3 V3	2772	.84	1.111
Total	8382	.97	1.078

Q2: Would use.

	N	Mean	Std. Deviation
1 V1	2851	1.20	1.009
2 V2	2759	.73	1.074
3 V3	2772	.77	1.092
Total	8382	.90	1.079

Figure S1

Mean acceptability of SC-based applications and perceived willingness to use them: Anticancer therapy (V1), Conversion of CO_2 emissions to biofuel (V2), and Industrial waste recycling (V3).

The figure shows Likert scale scores as numeric values: -2 for extremely unacceptable (Q1) or unlikely (Q2), -1 for somewhat unacceptable/unlikely, 0 for neither unacceptable/unlikely, nor acceptable/likely, 1 for somewhat acceptable/likely, and 2 for extremely acceptable/likely.

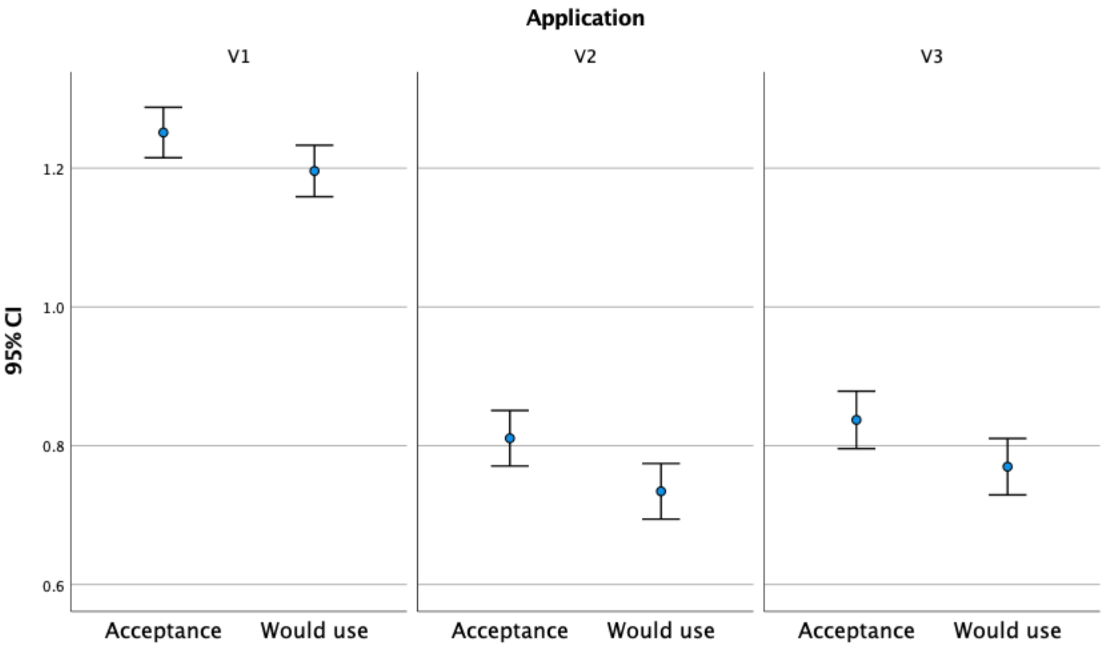

Table S5.
Synthetic cell applications acceptance per gender: Mean and Standard Deviation.

The table shows Likert scale scores as numeric values: -2 for extremely unacceptable (Q1) or unlikely (Q2), -1 for somewhat unacceptable/unlikely, 0 for neither unacceptable/unlikely, nor acceptable/likely, 1 for somewhat acceptable/likely, and 2 for extremely acceptable/likely.

	**F**		**M**	
	M	*SD*	*M*	*SD*
Anticancer therapy	1.25	0.98	1.25	1.01
Conversion of CO_2 emissions to biofuel	0.72	1.04	0.91	1.10
Industrial waste recycling	0.73	1.10	0.95	1.11

Figure S2.
Perceived acceptability of SC-based applications per gender.

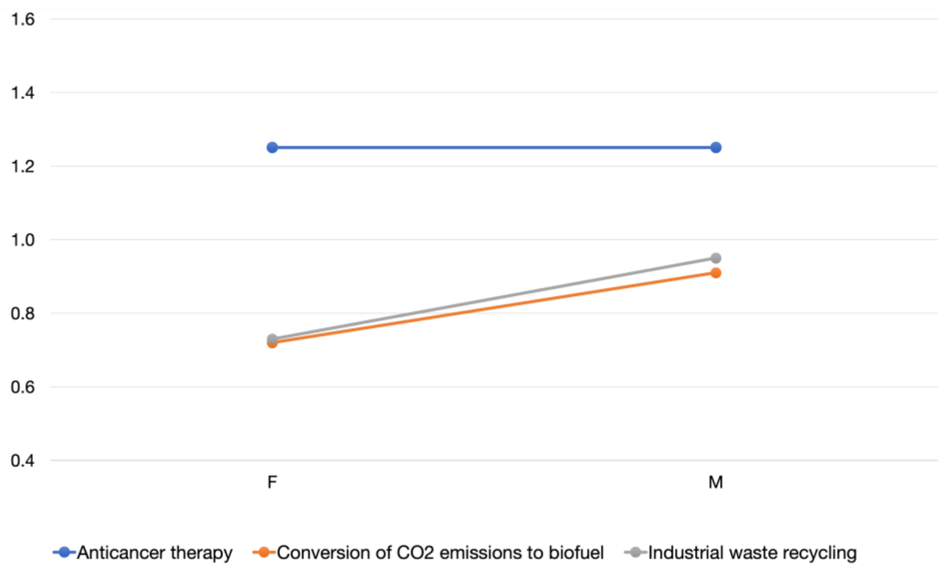

The figure shows Likert scale scores as numeric values: -2 for “extremely unacceptable”, -1 for “somewhat unacceptable”, 0 for “neither unacceptable, nor acceptable”, 1 for “somewhat acceptable”, and 2 for “extremely acceptable”.

Table S6.
Synthetic cell applications acceptance per age: Mean and Standard Deviation.

The table shows Likert scale scores as numeric values: -2 for "extremely unacceptable", -1 for "somewhat unacceptable", 0 for "neither unacceptable, nor acceptable", 1 for "somewhat acceptable", and 2 for "extremely acceptable".

	**18-24**		**25-34**		**35-44**		**45-54**		**55-64**		**65+**	
	M	*SD*	*M*	*SD*	*M*	*SD*	*M*	*SD*	*M*	*SD*	*M*	*SD*
Anticancer therapy	1.02	1.14	1.22	0.95	1.22	1.02	1.23	1	1.29	0.97	1.37	0.93
Conversion of CO_2 emissions to biofuel	0.78	1.08	0.74	1.09	0.78	1.05	0.89	1.08	0.83	1.03	0.82	1.09
Industrial waste recycling	0.89	1.17	0.82	1.13	0.76	1.1	0.79	1.11	0.78	1.1	0.96	1.08

Figure S3.
Perceived acceptability of SC-based applications per age.

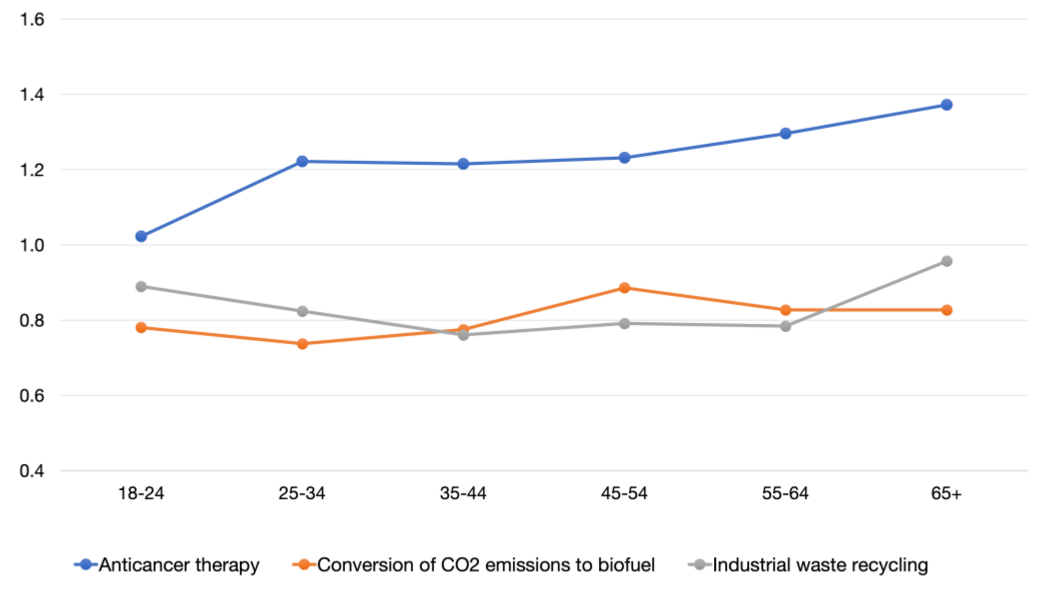

The figure shows Likert scale scores as numeric values: -2 for "extremely unacceptable", -1 for "somewhat unacceptable", 0 for "neither unacceptable, nor acceptable", 1 for "somewhat acceptable", and 2 for "extremely acceptable".

Table S7.
Synthetic cell applications acceptance per education level: Mean and Standard Deviation.

The table shows Likert scale scores as numeric values: -2 for “extremely unacceptable”, -1 for “somewhat unacceptable”, 0 for “neither unacceptable, nor acceptable”, 1 for “somewhat acceptable”, and 2 for “extremely acceptable”.

	**Low**		**Middle**		**High**	
	M	*SD*	*M*	*SD*	*M*	*SD*
Anticancer therapy	1.13	1.08	1.24	0.98	1.41	0.88
Conversion of CO_2 emissions to biofuel	0.67	1.19	0.78	1.03	1.00	0.98
Industrial waste recycling	0.69	1.16	0.83	1.09	1.03	1.06

Figure S4.
Perceived acceptability of SC-based applications per education level.

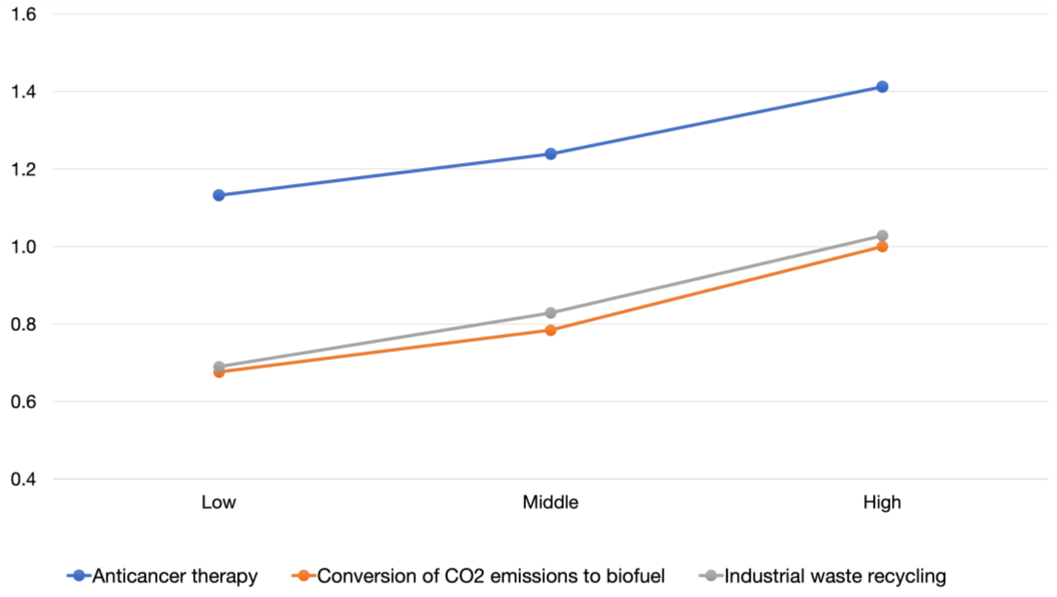

The figure shows Likert scale scores as numeric values: -2 for “extremely unacceptable”, -1 for “somewhat unacceptable”, 0 for “neither unacceptable, nor acceptable”, 1 for “somewhat acceptable”, and 2 for “extremely acceptable”.

Table S8.
Synthetic cell applications acceptance per religion: Mean and Standard Deviation.

The table shows Likert scale scores as numeric values: -2 for “extremely unacceptable”, -1 for “somewhat unacceptable”, 0 for “neither unacceptable, nor acceptable”, 1 for “somewhat acceptable”, and 2 for “extremely acceptable”.

	**Orthodox**		**Catholic**		**Muslim**		**Protestant**		**Non-religious**	
	M	*SD*	*M*	*SD*	*M*	*SD*	*M*	*SD*	*M*	*SD*
Anticancer therapy	1.23	0.98	1.26	1	1.33	0.97	1.39	0.92	1.32	0.95
Conversion of CO_2 emissions to biofuel	0.83	1.08	0.81	1.03	0.66	1.46	0.88	1.05	0.94	0.97
Industrial waste recycling	0.66	1.16	0.80	1.07	1.01	1.29	0.94	0.98	0.96	1.05

Figure S5.
Perceived acceptability of SC-based applications per religion.

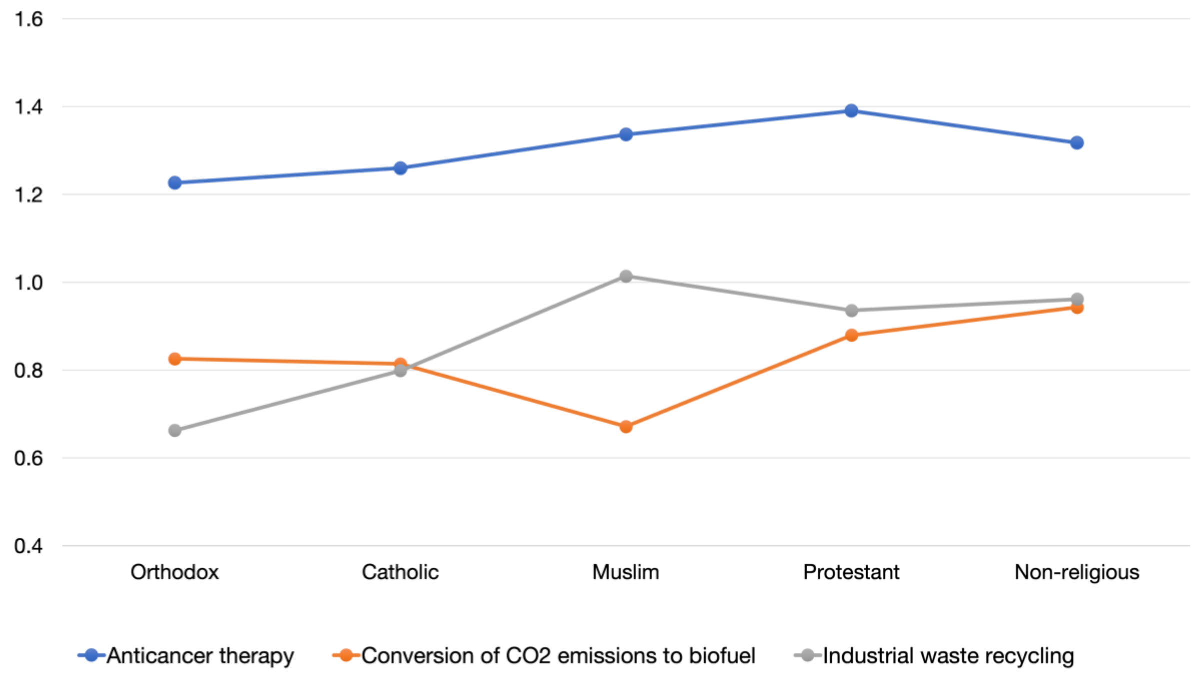

The figure shows Likert scale scores as numeric values: -2 for “extremely unacceptable”, -1 for “somewhat unacceptable”, 0 for “neither unacceptable, nor acceptable”, 1 for “somewhat acceptable”, and 2 for “extremely acceptable”.

Table S9.
Synthetic cell applications acceptance per country: Mean and Standard Deviation.

The table shows Likert scale scores as numeric values: -2 for "extremely unacceptable", -1 for "somewhat unacceptable", 0 for "neither unacceptable, nor acceptable", 1 for "somewhat acceptable", and 2 for "extremely acceptable".

	**CZ**		**DE**		**ES**		**FR**	
	M	*SD*	*M*	*SD*	*M*	*SD*	*M*	*SD*
Anticancer therapy	1.39	0.91	1.43	0.93	1.45	1.01	1.04	1.10
Conversion of CO_2 emissions to biofuel	0.68	0.98	0.81	1.01	1.10	1.06	0.70	1.05
Industrial waste recycling	1.01	1.01	0.82	1.07	1.09	1.05	0.50	1.20

	**GR**		**HU**		**IT**		**NL**	
	M	*SD*	*M*	*SD*	*M*	*SD*	*M*	*SD*
Anticancer therapy	1.23	0.91	1.45	1.01	1.26	0.90	1.07	1.06
Conversion of CO_2 emissions to biofuel	0.90	1.04	0.83	1.09	0.88	0.96	0.75	0.98
Industrial waste recycling	0.77	1.20	0.79	1.30	0.71	1.07	0.91	0.95

	**PL**		**RO**		**SE**		**TR**		**UK**	
	SD	*M*	*SD*	*M*	*SD*	*M*	*SD*	*M*	*M*	*SD*
Anticancer therapy	0.94	1.20	1.03	0.95	0.94	1.20	1.03	0.95	1.39	0.83
Conversion of CO_2 emissions to biofuel	0.99	0.87	1.09	0.73	0.99	0.87	1.09	0.73	0.96	1.00
Industrial waste recycling	1.07	0.82	1.12	0.85	1.07	0.82	1.12	0.85	0.95	0.94

Figure S6.
Perceived acceptability of SC-based anticancer therapy (scenario 1) per country.

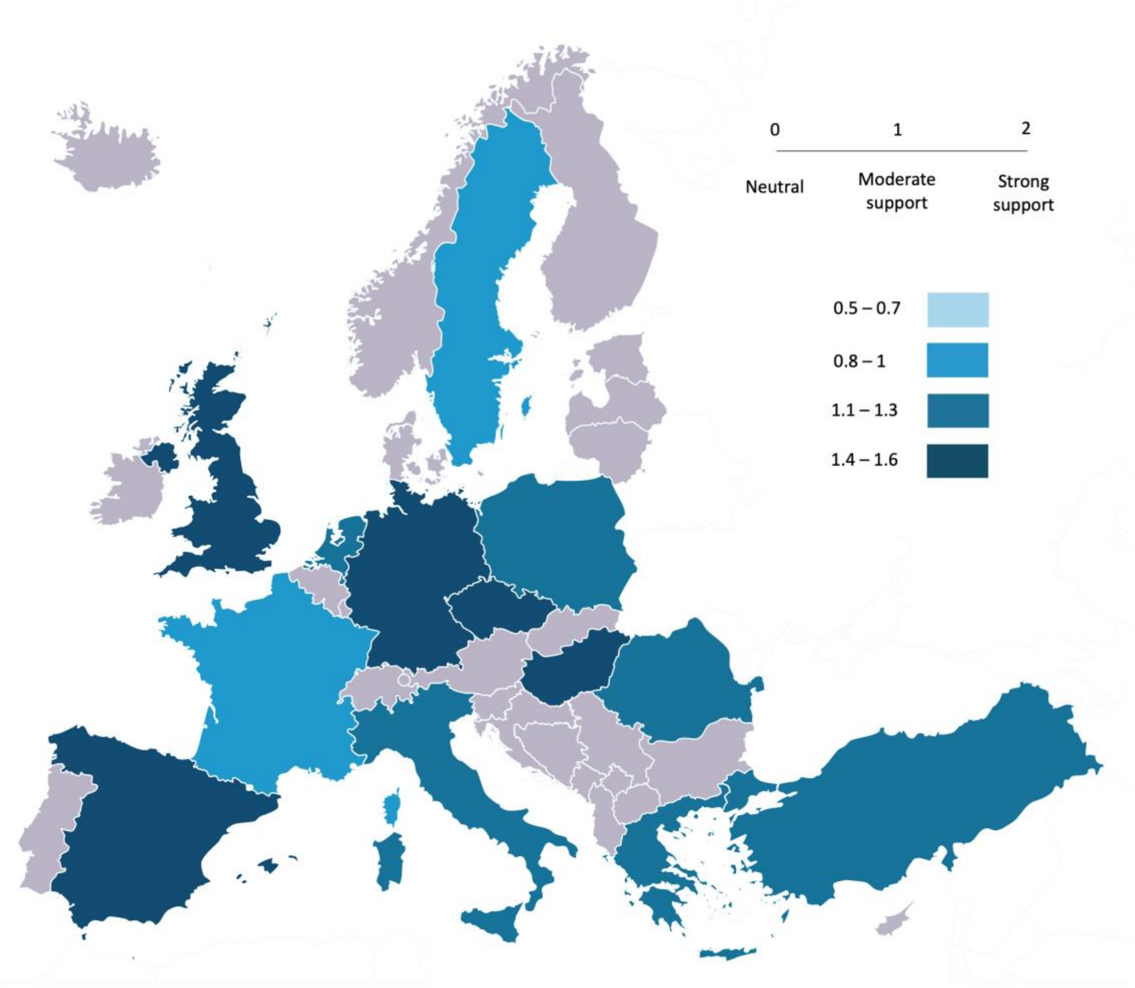

Created with MapChart.net, used under a CC BY license, with permission from MapChart.net.

The figure shows Likert scale scores as numeric values: -2 for “extremely unacceptable”, -1 for “somewhat unacceptable”, 0 for “neither unacceptable, nor acceptable”, 1 for “somewhat acceptable”, and 2 for “extremely acceptable”.

Figure S7.

Perceived acceptability of SC-based conversion of CO_2 emissions to biofuel (scenario 2) per country.

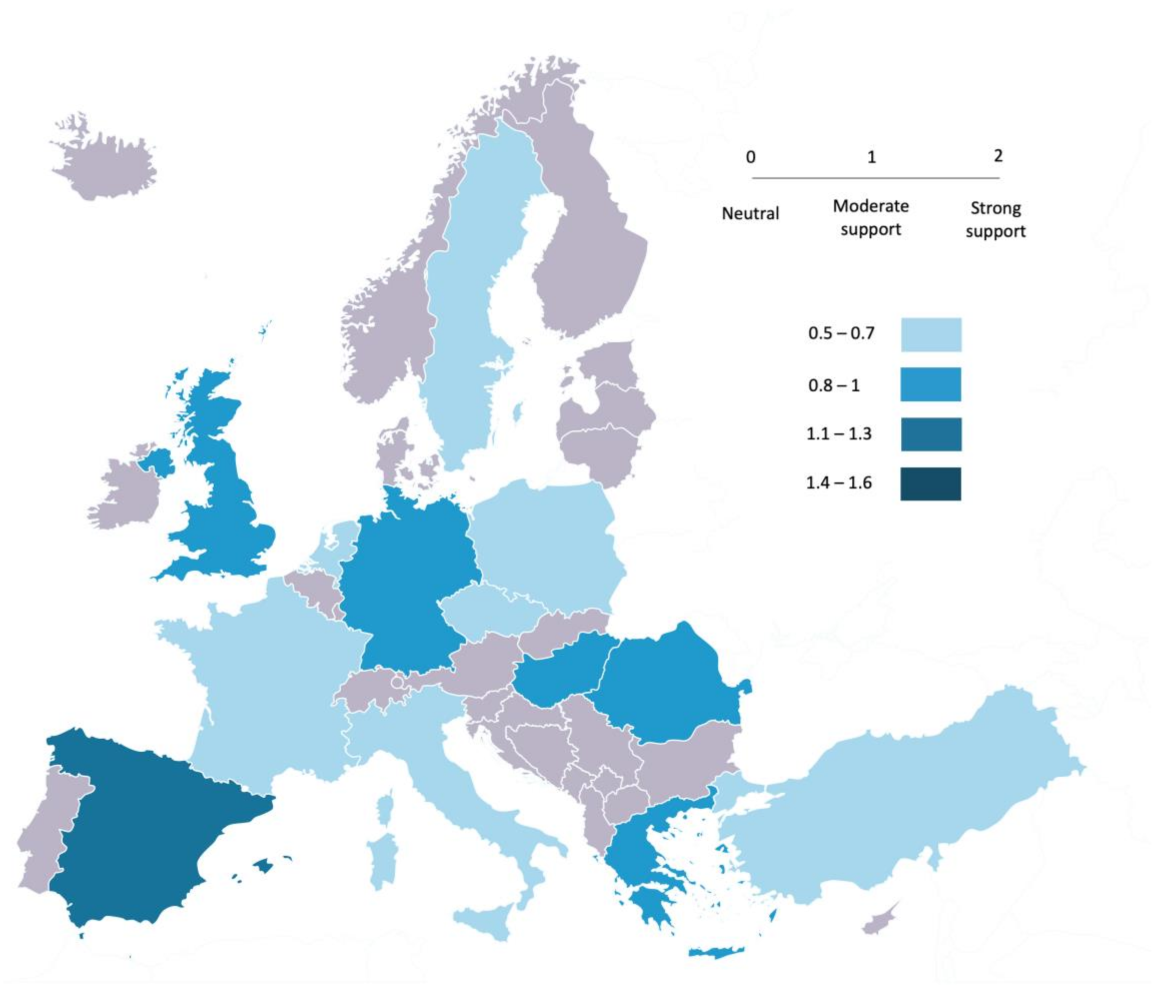

Created with MapChart.net, used under a CC BY license, with permission from MapChart.net.

The figure shows Likert scale scores as numeric values: -2 for “extremely unacceptable”, -1 for “somewhat unacceptable”, 0 for “neither unacceptable, nor acceptable”, 1 for “somewhat acceptable”, and 2 for “extremely acceptable”.

Figure S8.
Perceived acceptability of SC-based industrial waste recycling (scenario 3) per country.

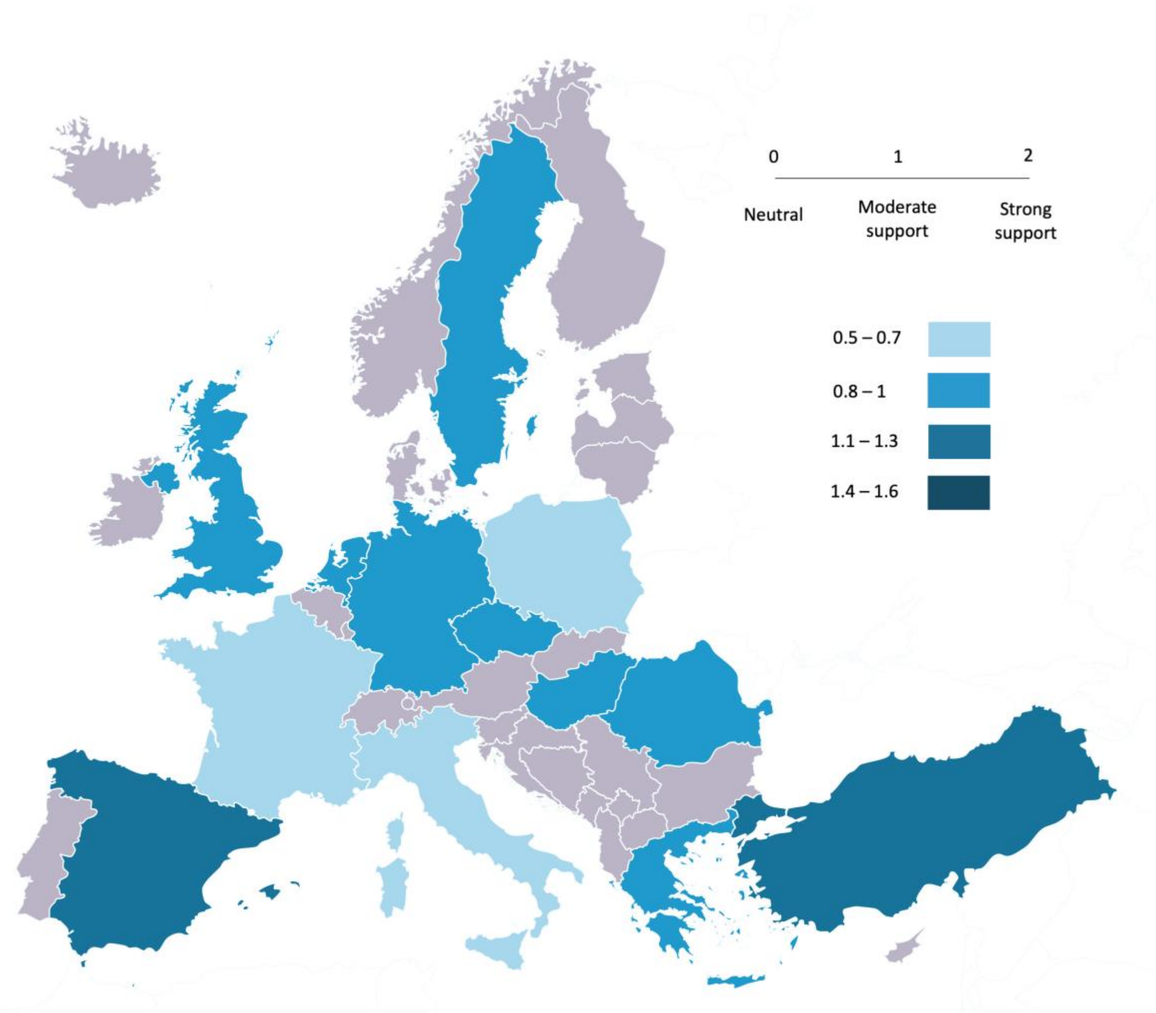

Created with MapChart.net, used under a CC BY license, with permission from MapChart.net.

The figure shows Likert scale scores as numeric values: -2 for “extremely unacceptable”, -1 for “somewhat unacceptable”, 0 for “neither unacceptable, nor acceptable”, 1 for “somewhat acceptable”, and 2 for “extremely acceptable”.

Figure S9.

Perceived acceptability of SC-based applications: Decision Tree.

Bars in the boxes show Likert scale score distribution.

a. All nodes.

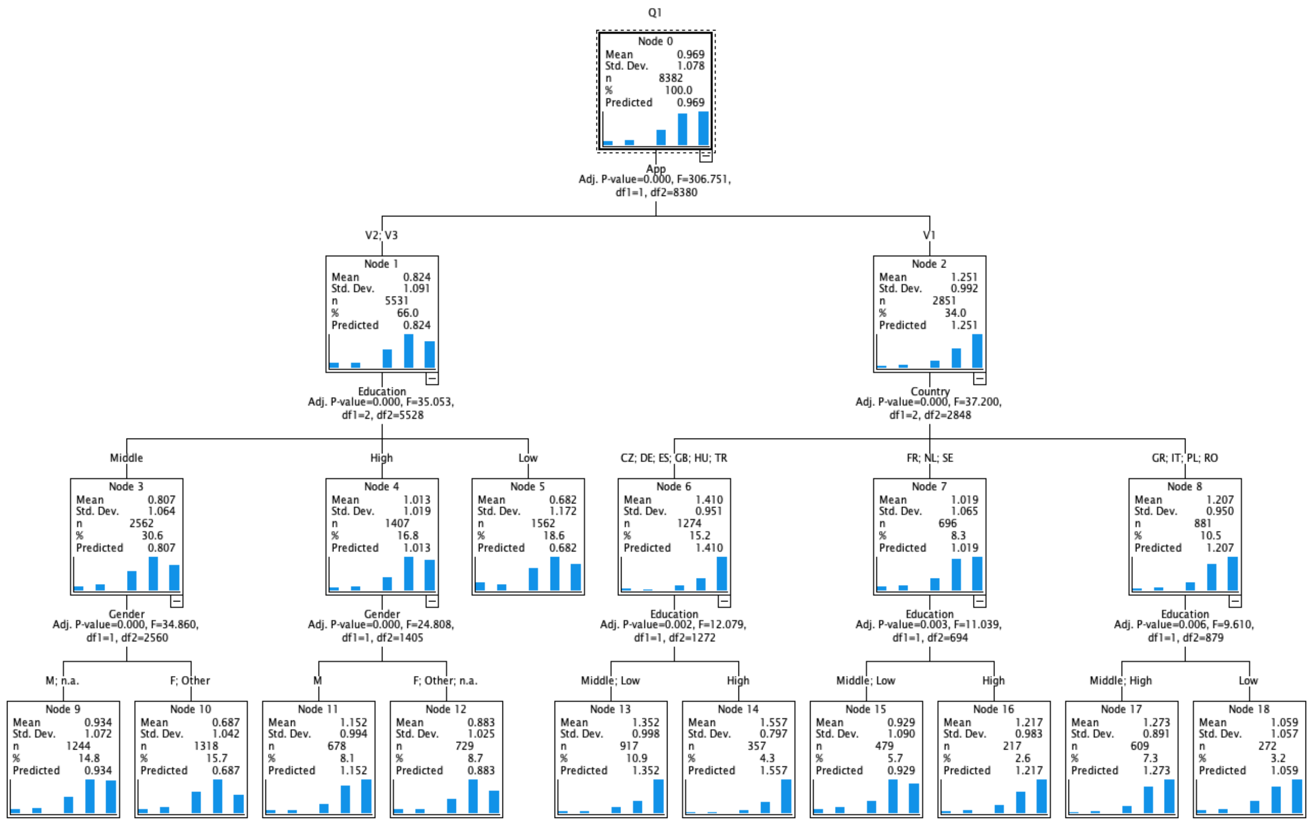

The figure shows Likert scale scores as numeric values: -2 for “extremely unacceptable”, -1 for “somewhat unacceptable”, 0 for “neither unacceptable, nor acceptable”, 1 for “somewhat acceptable”, and 2 for “extremely acceptable”.

b. Detail: Node 1 (vignettes 2 and 3: CO_2 conversion and waste recycling) tree part.

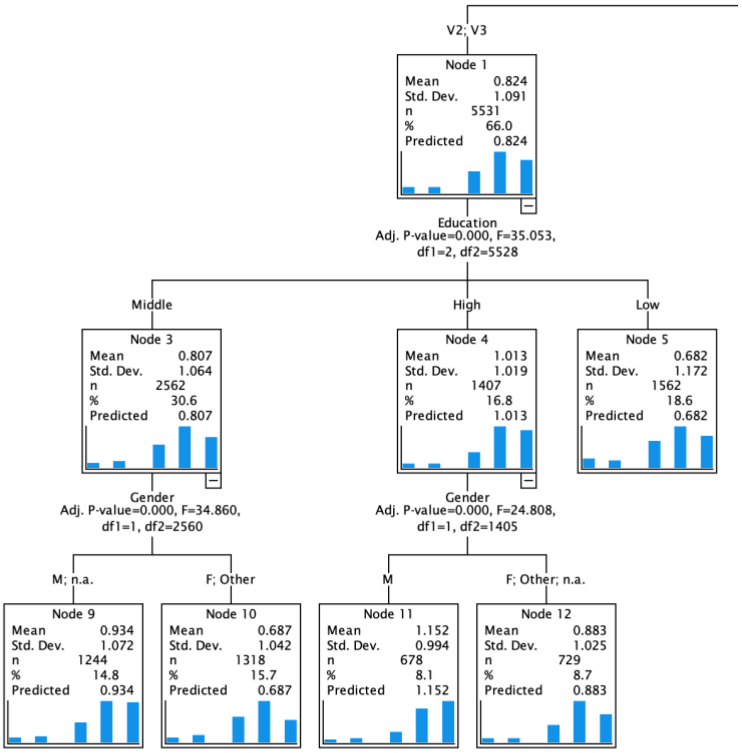

c. Detail: Node 2 (vignette 1: anticancer therapy) tree part.

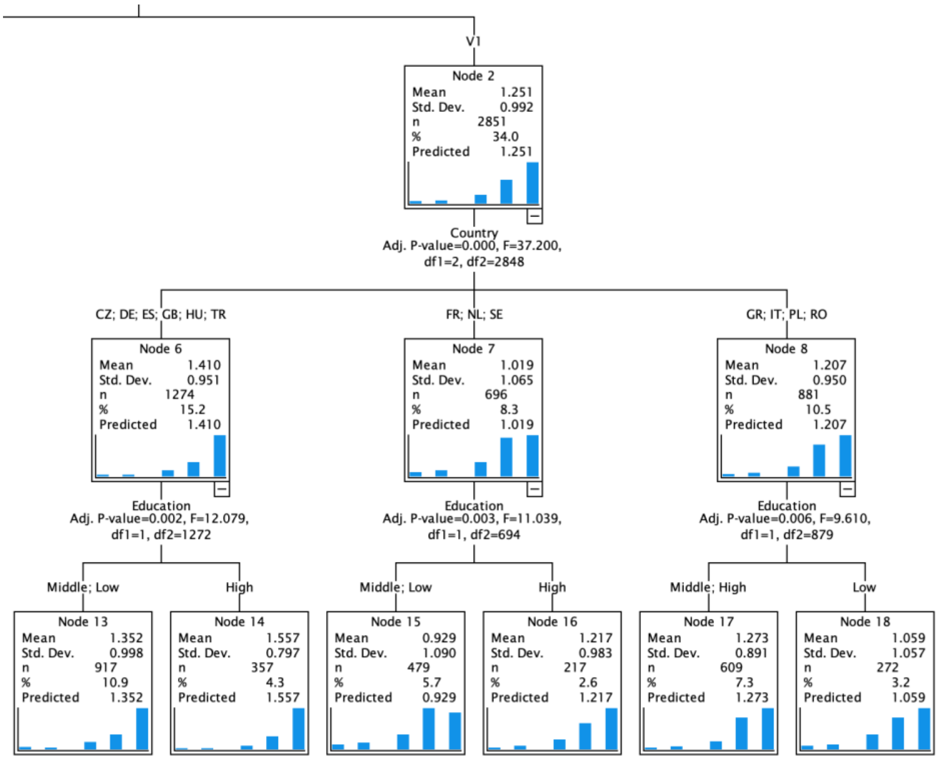

Figure S10.
Perceived willingness to use SC-based applications: Decision Tree.
Bars in the boxes show Likert scale score distribution.
a. All nodes.

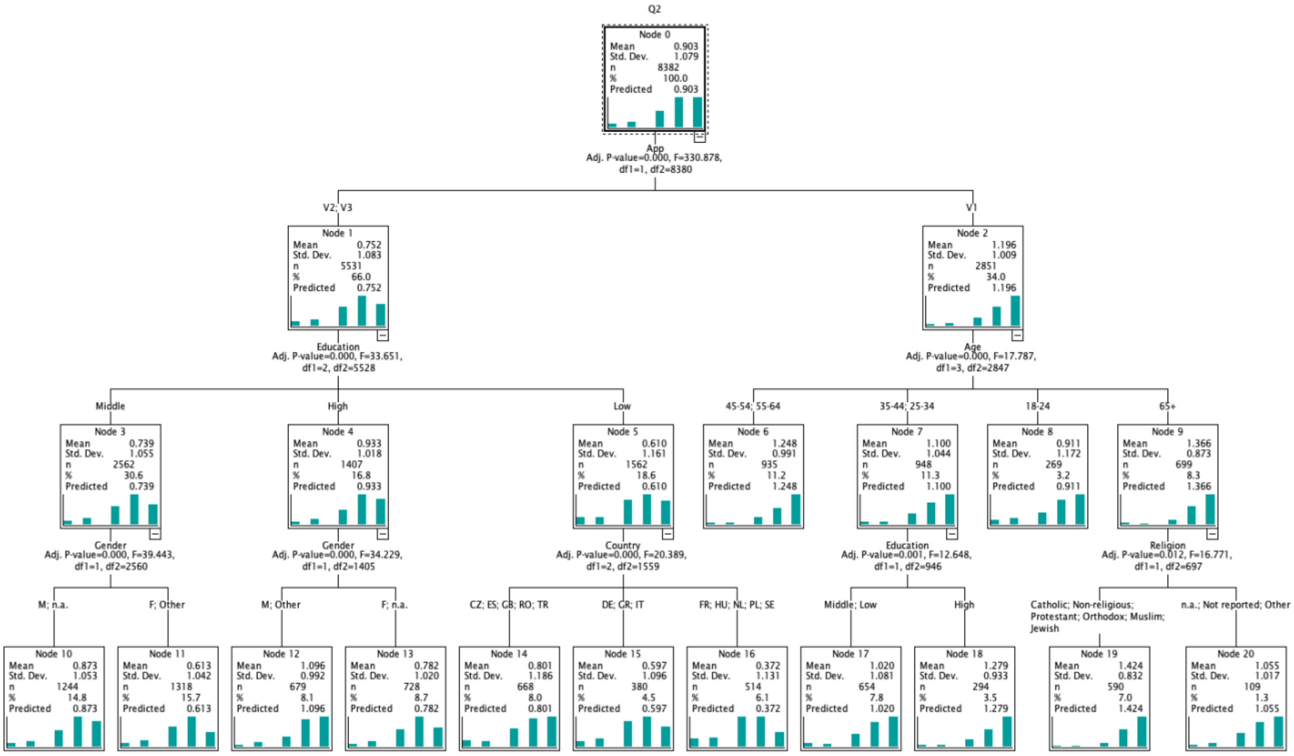

The figure shows Likert scale scores as numeric values: -2 for “extremely unlikely”, -1 for “somewhat unlikley”, 0 for “neither unlikely, nor likely”, 1 for “somewhat likely”, and 2 for “extremely likely”.

b. Detail: Node 1 (vignettes 2 and 3: CO_2 conversion and waste recycling) tree part.

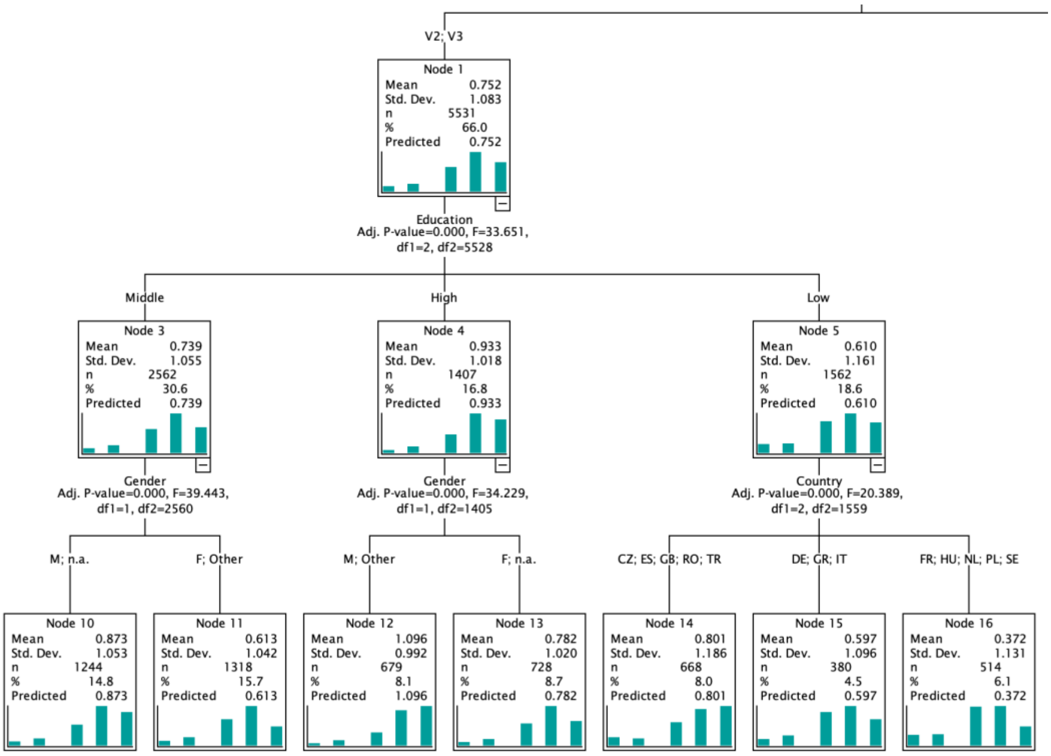

c. Detail: Node 2 (vignette 1: anticancer therapy) tree part.

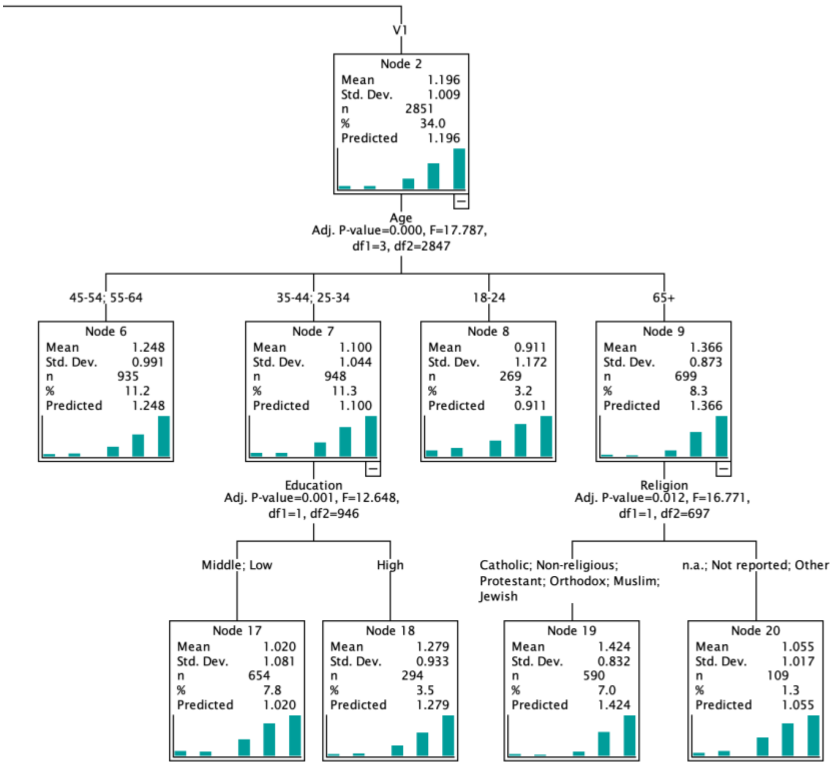

Figure S11.
Topic modeling process overview.

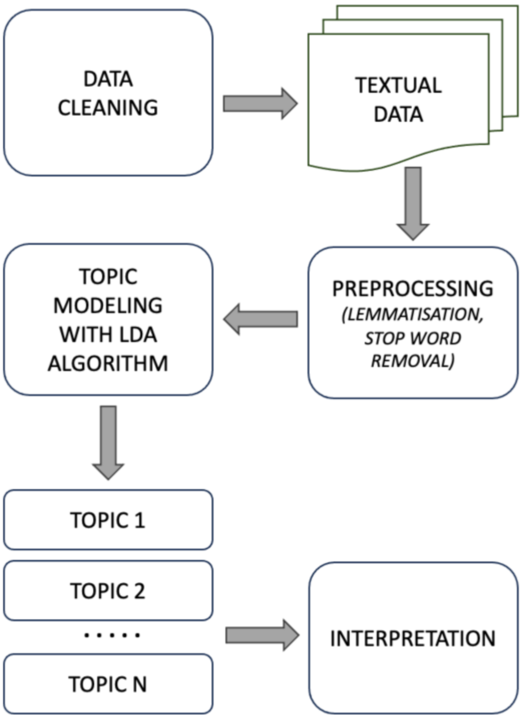

Table S10.
Topic modeling of the respondents' comments to scenario 1 (anticancer therapy).

The "topic" column contains a "label" description reflecting the author's interpretation of the topic as based on the top words and confirmed by typical entries per topic. Weight indicates relative prominence of the topics within the model (numeric values depend on the number of iterations). LL/T (log-likelihood per token) represents the overall model fit.

Topic	Weight	% of texts	Top Words
1. a decision to use one's chance to live, a hope	0.35	35%	chance - cancer - live - cure - hope - good - disease -treatment - die
2. an alternative therapy when the old treatment is no longer effective	0.18	18%	treatment - work - therapy - option - method - longer - lose - alternative - effective
3. saving or prolonging one's life is worth it	0.14	14%	life - save - health - worth - thing - important - chance - prolong - death
4. a personal choice in a hard situation	0.10	10%	decision - make - decide - agree - choice - future - situation- story - patient
5. side effects: could artificial cells affect healthy cells?	0.08	8%	cell - effect - healthy - side - cancer - artificial – treatment - affect - synthetic
6. trust in doctors and the progress of medical research	0.08	8%	science - trust - doctor - research - medicine - advance - medical - technology - progress
7. information is needed on possible side effects	0.05	5%	effect - side - treatment - alive - agree - stay - research - information - therapy
8. avoiding chemotherapy	0.03	3%	cancer - die - ill - year – chemotherapy - treat - chemo - read - avoid
LL/T=	*-6.63*		

Table S11.
Topic modeling of the respondents' comments to scenario 2 (conversion of CO_2 emissions to biofuel).

The "topic" column contains a description reflecting the author's interpretation of the topic as based on the top words and confirmed by typical entries per topic. Weight indicates relative prominence of the topics within the model (numeric values depend on the number of iterations). LL/T (log-likelihood per token) represents the overall model fit.

**Topic**	**Weight**	**% of texts**	**Top Words**
1. a good environmental idea for the planet	0.32	42.10%	good - environment - agree - idea - solution - planet - technology - sound - decision
2. reduces greenhouse gas while producing energy	0.22	28.95%	greenhouse - reduce - effect - gas - biofuel - technology - energy - good - pollution
3. one needs more information on synthetic cells to make decision	0.12	16.67%	cell - information - make - artificial - decision - artificially - synthetic – thing - give
4. the need to solve the global warming problem	0.10	13.84%	climate - problem - change - global - warming - solve - environmental – protection - situation
LL/T=	-6.52		

Table S12.
Topic modeling of the respondents' comments to scenario 3 (industrial waste recycling).

The "topic" column contains a description reflecting the author's interpretation of the topic as based on the top words and confirmed by typical entries per topic. Weight indicates relative prominence of the topics within the model (numeric values depend on the number of iterations). LL/T (log-likelihood per token) represents the overall model fit.

**Topic**	**Weight**	**% of texts**	**Top Words**
1. an environmentally friendly waste recycling technology	0.42	52.50%	good - waste - environment - technology - sound - environmentally - recycle - idea - friendly
2. turns waste into a useful material without releasing greenhouse gas	0.24	30.00%	waste - gas - greenhouse - material - chemical - medicine - plastic - produce - release
3. risk/benefit information needed on this technology	0.14	17.50%	make - decision - information - agree - technology - opinion - cell - risk - benefit
LL/T=	*-6.41*		

Figure S12.

Visualization of integrated TM and SA: SC anticancer therapy scenario.

The centrality of the box and the intensity of yellow reflect topic weight (prominence) vs. other topics. Word size represents relative frequency of the words within the topic. Additionally, word colors indicate sentiment: green for positive, red for negative, and blue for neutral.

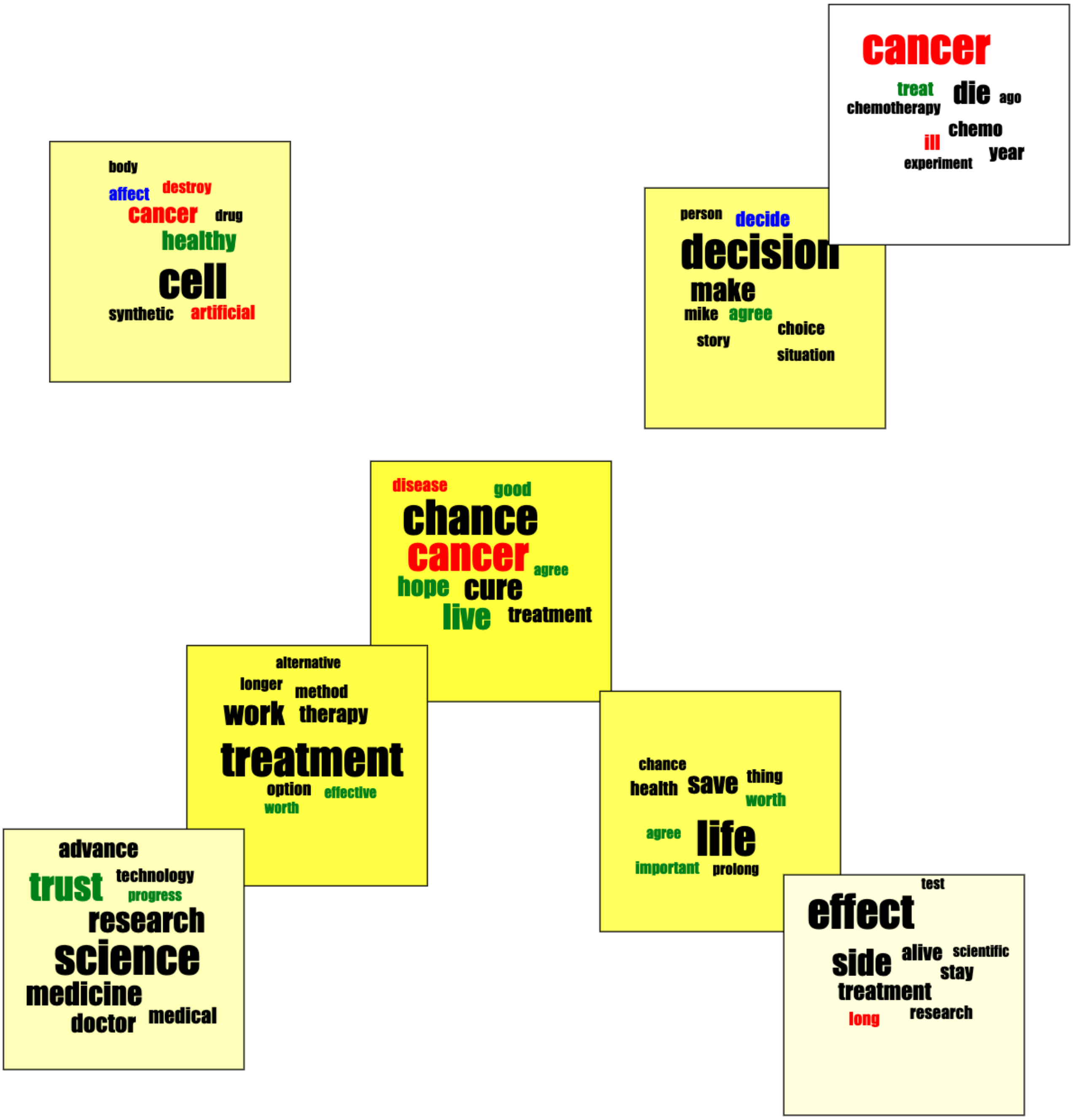

Figure S13.
Visualization of integrated TM and SA: SC-based conversion of CO_2 emissions to biofuel.

The centrality of the box and the intensity of yellow reflect topic weight (prominence) vs. other topics. Word size represents relative frequency of the words within the topic. Additionally, word colors indicate sentiment: green for positive, red for negative, and blue for neutral.

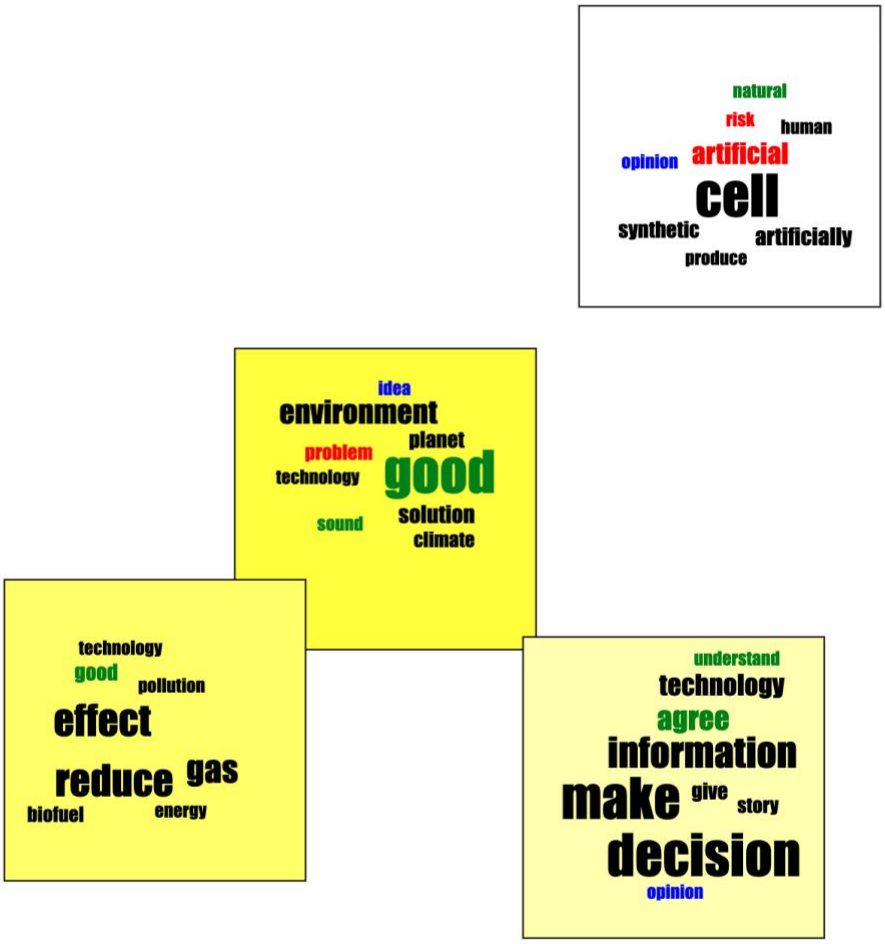

Figure S14.
Visualization of integrated TM and SA: CS-based industrial waste recycling scenario.

The centrality of the box and the intensity of yellow reflect topic weight (prominence) vs. other topics. Word size represents relative frequency of the words within the topic. Additionally, word colors indicate sentiment: green for positive, red for negative, and blue for neutral.

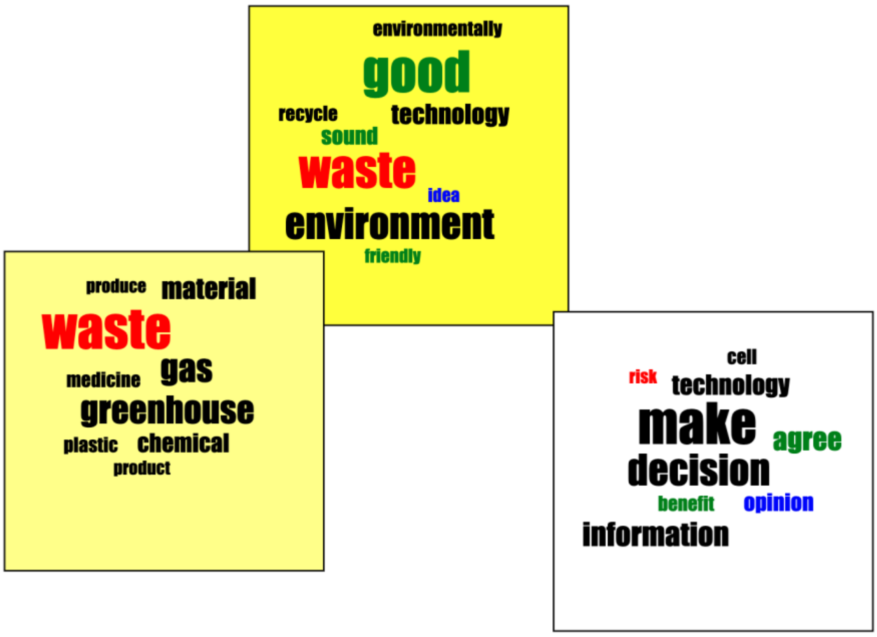

Supplement: S1 File — S1 Table. Demographics/Population characteristics. Abbreviations here and below: Czech Republic (CZ), Germany (DE), Spain (ES), France (FR), Greece (GR), Hungary (HU), Italy (IT), Netherlands (NL), Poland (PL), Romania (RO), Sweden (SE), Turkey (TR), United Kingdom (UK). S2 Table. Criteria for excluding survey data from statistical and/or textual analysis. S3 Table. Sample size per country before and after removing invalid entries. S4 Table. Mean acceptability of SC-based applications. Anticancer therapy (V1), Conversion of CO2 emissions to biofuel (V2) and Industrial waste recycling (V3). S5 Table. Synthetic cell applications acceptance per gender: Mean and Standard Deviation. Synthetic cell applications acceptance per gender: Mean and Standard Deviation. S6 Table. Synthetic cell applications acceptance per age category: Mean and Standard Deviation. S7 Table. Synthetic cell applications acceptance per education level: Mean and Standard Deviation. S8 Table. Synthetic cell applications acceptance per religion: Mean and Standard Deviation. S9 Table. Synthetic cell applications acceptance per country: Mean and Standard Deviation. S10 Table. Topic modeling of the respondents’ comments to scenario 1 (anticancer therapy). The “topic” column contains a “label” description reflecting the author’s interpretation of the topic as based on the top words and confirmed by typical entries per topic. Weight indicates relative prominence of the topics within the model (numeric values depend on the number of iterations). LL/T (log-likelihood per token) represents the overall model fit. S11 Table. Topic modeling of the respondents’ comments to scenario 2 (conversion of CO2 emissions to biofuel). The “topic” column contains a description reflecting the author’s interpretation of the topic as based on the top words and confirmed by typical entries per topic. Weight indicates relative prominence of the topics within the model (numeric values depend on the number of iterations). LL/T (log [file pone.0319337.s001.pdf]
