# Supplementary material for: Correction: Public attitudes to potential synthetic cells applications: Pragmatic support and ethical acceptance
Source: PLoS One. 2026 May 29;21(5):e0350696. doi: 10.1371/journal.pone.0350696 (PMC13221053; doi:10.1371/journal.pone.0350696)
Supplement: S2 File — (PDF) [file pone.0350696.s002.pdf]

RESEARCH ARTICLE

Public attitudes to potential synthetic cells applications: Pragmatic support and ethical acceptance

Olga Rook[1,2*], **Hub Zwart**[2], **Marileen Dogterom**[1]

1 Department of Bionanoscience, Kavli Institute of Nanoscience, Delft University of Technology, Delft, the Netherlands, **2** Erasmus School of Philosophy, Erasmus University Rotterdam, Rotterdam, the Netherlands

* o.rook@tudelft.nl

Check for updates

OPEN ACCESS

Citation: Rook O, Zwart H, Dogterom M (2025) Public attitudes to potential synthetic cells applications: Pragmatic support and ethical acceptance. PLoS One 20(2): e0319337. https://doi.org/10.1371/journal.pone.0319337

Editor: Alberto Molina Pérez, University of Granada: Universidad de Granada, SPAIN

Received: September 16, 2024

Accepted: January 30, 2025

Published: February 27, 2025

Copyright: © 2025 Rook et al. This is an open access article distributed under the terms of the Creative Commons Attribution License, which permits unrestricted use, distribution, and reproduction in any medium, provided the original author and source are credited.

Data availability statement: The anonymized original data are publicly available via the Open Science Framework (https://doi.org/10.17605/OSF.IO/2H54A).

Funding: This study is part of Building a Synthetic Cell (BaSyC), a Gravitation grant program funded by the Netherlands Organization for Scientific Research (NWO). Grant reference number: 024.003.019. The funders had no role in the study design, data collection and

Abstract

Synthetic cells constructed bottom-up represent a novel direction in Synthetic Biology. It has the potential to deepen the scientific understanding of life and, in the longer run, to open up new pathways for medical and environmental applications. Mapping preliminary public attitudes towards emerging technologies is an important step to further societal discussion and stakeholder participation. We conducted a vignette survey with nationally representative samples from 13 European countries (Czech Republic, France, Germany, Greece, Hungary, Italy, the Netherlands, Poland, Romania, Spain, Sweden, Turkey, and UK; $N = 8,382$) to explore public attitudes towards prospective synthetic cell technologies, such as anticancer therapy, CO_2 emissions conversion to biofuel, and industrial waste recycling. Using data-driven techniques, we built a decision tree model of the factors affecting participants' attitudes and summarized the prevalent themes behind one's motivation. Our findings suggest substantial public support for prospective synthetic cell applications in the societally beneficial fields, most notably in healthcare.

Introduction

Synthetic cell (SC) research is part of a larger field of synthetic biology (SynBio), which is emerging as an important direction in biotechnology. In SynBio, multidisciplinary science meets engineering to re-design existing biological systems or components and build new ones [1–4]. The engineering approach is used to obtain desired functions or specifications and serves two main goals: to gain a detailed understanding of biological mechanisms, and to produce useful bio-based applications and materials [5]. Designing autonomous self-reproducing SCs should first of all address important questions concerning how life works, but also allow for practical applications [6]. In a top-down approach to SCs, a living bacterium's genome is replaced with a minimized synthetic one, reprogramming the cell and making space for designed elements and functions [7–9]. In contrast, the bottom-up approach aims to construct all elements of functional self-sustaining SCs from scratch, using non-living molecular components (natural or artificial), and reconstituting transition of matter into a living state [10,11]. This endeavor has potential to create new forms of life and may be considered a "threshold technology" with unique benefits and concerns [12,13]. Bottom-up SCs, therefore, entail a more radical version of SynBio, an ontological experiment even [14].

analysis, decision to publish, or preparation of the manuscript.

Competing interests: The authors have declared that no competing interests exist.

While the direct goal of bottom-up SCs is to shed light on the mechanisms and origin of life, this ground-breaking research may lead to novel bio-compatible and sustainable technologies in the near future. Those innovations could help address global problems (e.g., environmental or healthcare issues) [6,15–17], but might also bring about unknown ecological, societal, and ethical consequences. An anticipatory approach is essential to assess and decrease possible risks and to maximize benefits [18]. This includes not only standard risk assessment and regulatory strategies, but also citizen participation in SCs discussion [19,20]. Exploratory studies such as the current survey could set the ground for further steps in this direction.

To our knowledge, no studies so far specifically addressed public attitudes to SCs or potential SC-based technologies. Research on the public attitudes towards SynBio provides us with general background and insights into how people view the promises and risks of the emerging SynBio technologies both in general and in specific fields [21]. For example, medical, environmental and biofuel applications had typically been viewed as acceptable [22–28], whereas (agri)food applications less so [24,26,29]. How would publics react to technologies involving SCs? Would the creation and use of artificially-made cellular life mean crossing an ethical threshold? The existing SynBio studies offer limited and seemingly contradictory cues here. In a US focus group study, participants expressed negative reactions to the idea of "constructing" and releasing into one's body or into environment something that had not existed in it before [30]. In a subsequent telephone survey, more than a quarter of respondents agreed with a statement that "it is morally wrong to create artificial life" [24]. Conversely, in a large recent Australian survey, an entirely artificially designed pseudo-organism received the strongest public support (and only 3% of responses with intrinsic critical claims) compared to known species that had been genetically edited or engineered [31,32]. Likewise, in a 2020 Chinese survey on SynBio food applications, the respondents were less sensitive to the use of artificial genes than genes from other species [33]. All this suggests that one cannot automatically transfer insights on public attitudes towards SynBio to SCs. Synthetic, artificial cells and their applications warrant research as a distinct technology.

This study presents the findings on public acceptance of potential SC-based technologies from a cross-national online survey ($N = 8{,}382$). The survey with nationally representative population samples from 13 European countries (Czech Republic, France, Germany, Greece, Hungary, Italy, the Netherlands, Poland, Romania, Spain, Sweden, Turkey, and UK) was conducted in 13 languages. Each participant was randomly assigned to one of the three vignettes (scenarios) featuring "an imaginary story" placed somewhere in the future and involving SC applications: an anticancer treatment, a conversion of power plant CO_2 emissions to biofuel, and industrial waste recycling (Fig 1). These technically plausible potential applications were inspired by recent SynBio research [16,34,35] and focus on the fields prioritized by the public in the above-mentioned studies of SynBio perceptions. There was no emphasis on technical features, and no educational component: partly because such technologies do not (yet) exist and partly to assess, whether mentioning artificial/synthetic versions of biological units such as cells would evoke concerns on their own, rather than due to specific detail. Our vignettes embedded questions in practical context, which is appropriate for exploring attitudes rather than conceptual evaluation. The participants had to report whether they found acceptable the decision made in the story to use a SC-based application and whether they would have made the same decision. The questions thus involved normative and decision-making aspects pertaining to an individual (therapy), an industrial regulatory body (industrial application) and to government (CO_2 emissions conversion). All scenarios presented novel technologies where the risks are not fully calculable, and decision-making involves some uncertainty.

The survey allowed us to assess the initial public attitudes towards the SCs and their usage in societally relevant areas, to understand which potential applications might gain most public

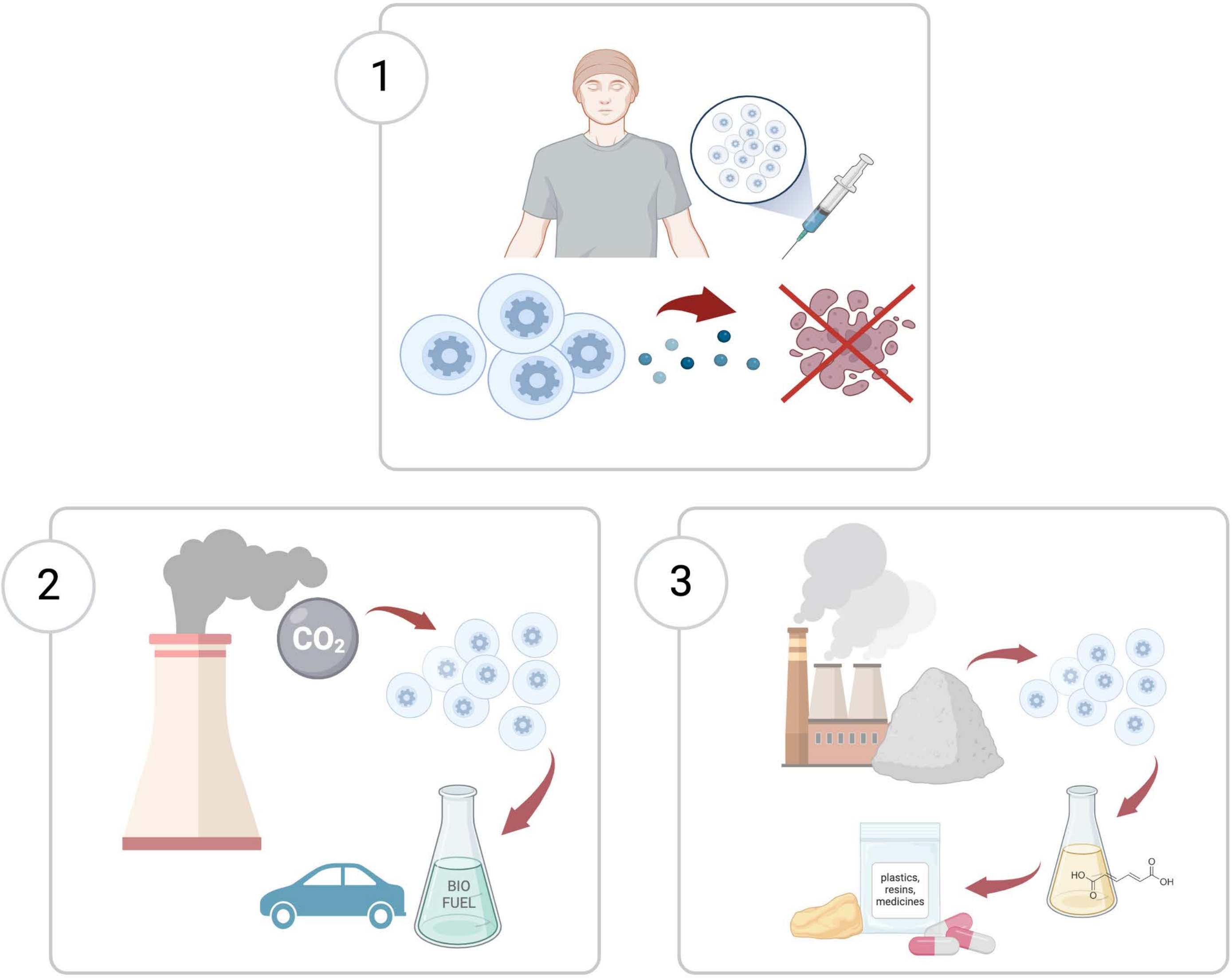

Fig 1. Survey vignettes. 1. Anticancer therapy with SCs. 2. Conversion of power plant CO_2 emissions to biofuel using a SCs installation. 3. Industrial waste recycling into a valuable chemical using SCs. Created with BioRender.com.

https://doi.org/10.1371/journal.pone.0319337.g001

support, to explore individual and cultural characteristics associated with these attitudes and review the motivation behind responses. This exploratory study may be regarded as a pre-consultation of the publics and a preparation for a more in-depth dialogue with societal stakeholders. Our findings may be relevant for scientists, public organizations, media, entrepreneurs, and policy makers involved in the biotechnology-related decision-making, regulation, and legislation.

Methodology

Sampling and data collection

We conducted a cross-sectional online survey in Czech Republic, France, Germany, Greece, Hungary, Italy, the Netherlands, Poland, Romania, Spain, Sweden, Turkey, and UK

(N = 8,382; 590-740 respondents per country). The countries were selected to represent different regions and cultural traditions across Europe. Survey participants were recruited by the panel agency Bilendi (ISO 20252-certified) from their panels in Western Europe and their partners' panels in Eastern Europe, Greece, and Turkey. All participants received a small remuneration for their time in the form of "points" exchangeable for gifts in the online panel shop. Quotas on gender, age and education distributions ensured representativeness of each country's adult national population (the participants' socio-demographic characteristics per country are presented in S1 Table in online Supplementary Information). Individuals younger than 18 years old were not eligible for the survey and were additionally screened out. The data were collected in Qualtrics between July 3 and September 1, 2023. A total of 8,413 individuals took part in the survey (excluding incomplete entries and obvious speeders). The survey took 3.4 minutes on an average to complete. Several invalid entries were removed from the dataset resulting in 8,382 responses (S2 Table provides an overview of the exclusion criteria and invalid entries; the identifiers of the entry numbers are anonymized).

Research design

The participants were informed that the goal of the study was to examine the attitudes people may hold about possible future technologies based on synthetic cells. After providing informed consent, the participants reported their gender, age category, education and, optionally, religion. Then they were presented with "an imaginary story" placed a few years from now. Each respondent was randomly assigned to one of the three vignettes featuring a potential technology. On a 5-point Likert scale, respondents reported whether they (1) found the decision in the story to use a SC-based application acceptable, and (2) if they would make the same decision in the same situation. They were also invited to comment why they agreed or disagreed with the decision made in the story. The Likert scale scores in the dataset were assigned numeric values: -2 for extremely unacceptable (Q1) or unlikely (Q2), -1 for somewhat unacceptable/unlikely, 0 for neither unacceptable/unlikely, nor acceptable/likely, 1 for somewhat acceptable/likely, and 2 for extremely acceptable/likely.

Vignettes and questionnaire

The vignettes featuring hypothetical SCs applications were developed based on the recent SC and SynBio advances. The choice of applications was determined by the plausibility of their short-term development given the current state of research [16,34,35], and by restricting our focus to several societally beneficial fields that had already been prioritized by participants in previous studies of SynBio perceptions [22–28]. The selected vignettes were approved by several researchers in the SC field we consulted.

Each vignette presented SCs as the basis of a novel technology, stressing their human-made origin ("artificially designed", "specially designed") and stating their function in each application. The term "synthetic cells" was used in all vignettes. No educational content on SCs was added, since we aimed to assess the participants' most immediate attitudes, not affected by additional information or discussion. This would also allow evaluating how salient, ontologically disturbing, or ethically sensitive the idea of artificial cells may appear as such. We avoided technical language and performed Fleisch-Kinkaid readability tests to ensure that participants with lower education could easily understand all stories. To keep the vignette texts as emotionally neutral as possible and to avoid priming, we tested our wording for emotion and objectivity dimensions using LIWC linguistic software. The resulting vignettes were presented and discussed with the research community during a biannual national SC consortium conference, cross-validated using anonymous online form, and adjusted.

This procedure yielded the following scenarios of SC use, for which the participants had to evaluate the acceptability of the decision and the likelihood one would have made the same choice:

(1) "Mike has cancer. The therapy he received some years ago does not work for him any longer. His doctor informed him about a new treatment. Mike can get an injection of artificially created cells into the cancerous area. These synthetic cells can sense the cancer cells and destroy them by producing a drug. This should not affect healthy cells. Mike decides to undergo this therapy."

(2) "A European country wants to reduce the greenhouse effect. A new technology uses artificially designed cells to absorb greenhouse gas and turn it into biofuel. Units with such synthetic cells can be placed at factories and power plants. The government approves this technology."

(3) "A new technology can be used to break down some types of industrial waste. Specially designed synthetic cells can be placed at a factory to break down its waste and convert it into a chemical. No greenhouse gas is released during this process. The resulting chemical serves as raw material for making resins, plastic, and medicines. The authorities approve this technology."

The original questionnaire was designed in English. Translations into the other 12 languages were performed by native speakers, mostly fellow researchers. The translators received written instructions on the desired level of simplicity and neutrality of wording. Translations involved at least 2 native speakers for each language to verify the correctness of the translation and to make adjustments, when needed. The resulting texts were translated back into English using DeepL and GoogleTranslate, and any ambiguous or unclear passages were meticulously reviewed with native speakers. The full questionnaire and its translations are publicly available in the study data package at the OSF repository.

Analysis

The data exported from Qualtrics was organized with Microsoft Excel. Statistical analyses were carried out in SPSS (v. 27). Descriptive statistical analysis included group means comparison and frequency distribution. We also performed decision tree analysis. For topic modelling, we used ConText (v. 2.0) text analysis software developed at the University of Illinois Urbana Champaign [36,37] based on MALLET language toolkit LDA implementation [38]. Visualization of integrated topic modeling and sentiment analysis was conducted in ConText using MPQA Subjectivity Lexicon [39]. Word clouds were made in ATLAS.ti (v. 24.1.0). Additionally, we used manual lexicon-based quantitative and qualitative analysis to evaluate several underrepresented topics.

Decision trees

Having a large sample with multilevel categorical independent variables (which may have non-linear relations between them), only two dependent variables (ordinal ones), and no explicit hypotheses to test, we have chosen a data-driven approach to explore the survey data. Decision tree (DT) analysis is an ML technique which can capture complex and flexible relationships between variables in large datasets [40–43]. DT algorithms employ recursive partitioning and divide the sample into subgroups homogeneous with respect to the dependent variable [40]. In a sequence of decision steps (nodes) based on heuristic search through independent variables (predictors), the data is split into "branches". The sequence of predictors reflects the rules governing the data and provides a predictive model [40,42]. Overfitting,

when the model cannot generalize to new data, is a common concern of predictive models including decision trees [42]. Various strategies could be used to avoid it. We performed a DT analysis using CHAID algorithm. It defines the statistical significance of independent variables and splits the data based on chi-square test. The CHAID method uses pre-pruning reducing the risk of overfitting, is optimal to use with categorical independent variables, and had been tested to provide accurate results [44]. Independent variables loaded into the DT model included application, gender, age, education level, religion, and country. Dependent variables were the Likert scale scores for SC application acceptability and willingness to use.

Topic modeling

Topic modeling (TM) is a text mining technique that reveals underlying patterns of term (word) usage by clustering highly co-occurring words into topics [45]. To uncover prominent themes in the participants' comments, we implemented the LDA (Latent Dirichlet Allocation) algorithm: a generative unsupervised probabilistic NLP technique based on a Bayesian inference to estimate document-topic and topic-term distribution [46]. It assumes that each document within the corpus may include a mixture of topics, and each topic an overlapping combination of words. This approach appears well-suited for a survey, where participants could express mixed attitudes behind their choices. Short texts could be more challenging for TM. LDA was tested against the other TM methods with short texts (such as social media data, online comments, and reviews) and yielded the best results, along with the non-negative matrix factorization [47]. Using ConText TM software, we compared public reactions to the three scenarios featuring specific SC-based applications.

The cleaned data went through pre-processing such as lemmatization and stop-word removal (S1 Fig outlines the TM procedure). With TM methods, one needs to define the number of topics and the number of words per topic in advance, which typically requires exploration for the best fit. Our choice of topic number was guided by the overall topic interpretability, stability (identical or nearly identical word combination for all topics across multiple runs) and consistent topic weight (relative prominence). We used a term-centered approach to evaluate topic stability and tested it across 11 algorithm runs for each vignette-based sub-corpus. Defining 8 topics for the medical scenario, 4 for carbon emission conversion and 3 for waste recycling, with 9 words per topic, yielded meaningful topics with high term stability across runs. Increasing the number of topics (and also decreasing it, in case of therapy scenario) led to confusing and uninformative topics. We applied $\Sigma\alpha = 5$ (a parameter controlling topic distribution within a document) with 3,500 iterations. Log-likelihood per token (i.e., per word) was used to estimate the overall model fit.

Ethics

Ethics approval was granted by the Research Ethics Review Committee of the Erasmus School of Philosophy, Erasmus University Rotterdam (reference: ETH2223-0601). The online survey participants (panel members of a research panel agency) were presented with information about the study and provided informed consent before proceeding to the survey questions.

Results

Descriptive statistics

Most of the survey respondents expressed some degree of support for potential SC-based use featured in the vignettes. Strong acceptance ranged between 29-32% for CO_2 conversion and waste recycling and 52% for anticancer treatment (Fig 2). Together, moderate and strong acceptance for these applications amounted to 68-69% and 82%, respectively. Neutral

position was more often reported for CO_2 conversion (21%) and waste recycling (19%) and was 11% for SC-based therapy. Strongly negative attitudes were reported by 3 to 6% of respondents. Cumulatively, 7 to 12% of participants found SC-based applications either strongly or somewhat unacceptable. Willingness to use such applications remained consistent with the acceptability results, with a slight variation: 2-3% less respondents expressed strong support and 2-3% more chose a neutral answer (Fig 3).

We compared the mean acceptability scores for SC-based applications per demographic category and per country (for detail, see S5-S9 Tables and S2-S8 Figs). Mean scores per gender did not differ for medical application ($M = 1.25$; $SD = 0.98$ for females and 1.01 for males) but males had slightly higher mean scores for the other two applications (with $M = 0.91–0.95$ vs. 0.72–0.73; SD = 1.11, 1.10, 0.72 and 0.73, respectively).

Mean acceptability scores for SC-based therapy increased with age: from 1.02 for 18–24 age category ($SD = 1.14$) to 1.37 for 65 + age group ($SD = 0.93$). The mean score difference per age was less pronounced for other applications, ranging from 0.78 to 0.96. For participants' education, mean scores had shown a distinct pattern growing with the increase in education level. For medical application, the mean score increased from 1.13 in lower education category ($SD = 1.08$) to 1.41 in higher education group ($SD = 0.88$). For CO_2 conversion and waste

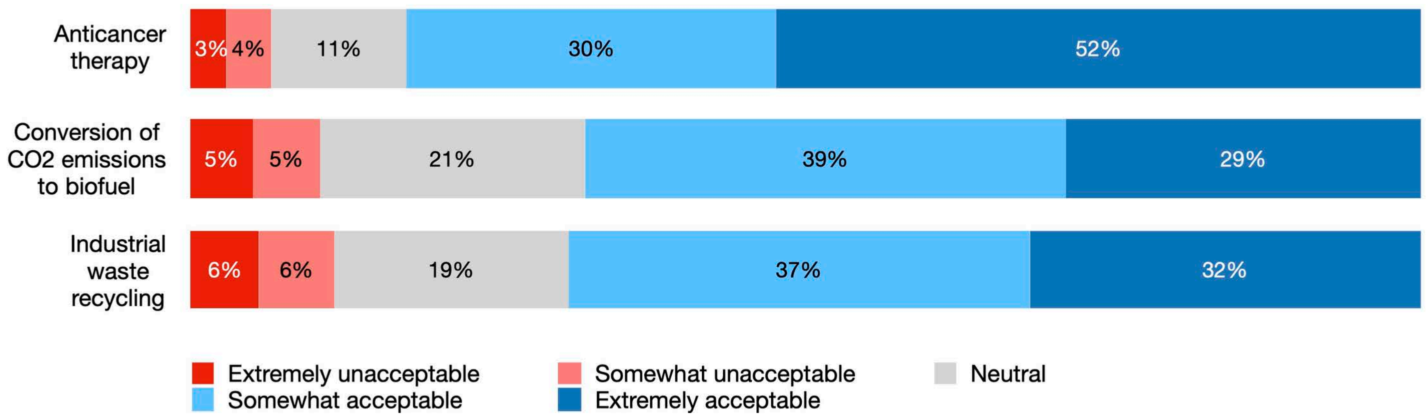

Fig 2. Acceptance of synthetic cell-based applications.

https://doi.org/10.1371/journal.pone.0319337.g002

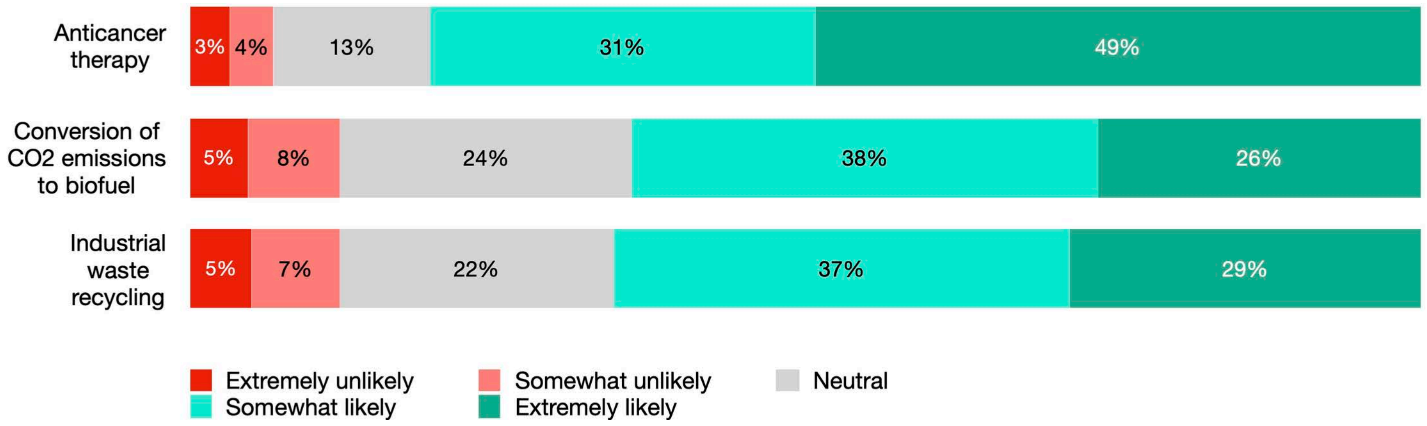

Fig 3. Willingness to use synthetic cell-based applications.

https://doi.org/10.1371/journal.pone.0319337.g003

recycling it increased from 0.67–0.69 (*SD* = 1.19 and 1.16) to 1.00 and 1.03 (*SD* = 0.98 and 1.06).

Religion was reported by 87.9% of the survey participants. The highest acceptance rates for SC-based therapy were reported by Protestant respondents (*M* = 1.39; *SD* = 0.92), followed by Muslim and non-religious participants. Muslim and Orthodox Christian respondents had, respectively, the highest and the lowest mean acceptance for SC-based industrial waste recycling (*M* = 1.01; *SD* = 1.29 vs. *M* = 0.66; *SD* = 1.16). For CO_2 conversion, the acceptance was the highest for non-religious and Protestant participants (*M* = 0.94; *SD* = 0.97 and *M* = 0.88; *SD* = 1.05) and the lowest among the Muslim respondents (*M* = 0.66; *SD* = 1.46).

At a country level (Fig 4), the highest acceptance scores for a SC-based therapy were reported in Spain and Hungary (both *M* = 1.45; *SD* = 1.01), followed by Germany (*M* = 1.43; *SD* = 0.93). The lowest scores were reported in Sweden (*M* = 0.95; *SD* = 1.04), followed by France (*M* = 1.04; *SD* = 1.10) and the Netherlands (*M* = 1.07; *SD* = 1.06). Spain had the highest scores for both CO_2 conversion (*M* = 1.10; *SD* = 1.06) and waste recycling (*M* = 1.09; *SD* = 1.05). The second highest acceptance rate for industrial waste recycling was in Turkey (*M* = 1.06; *SD* = 1.28), and for CO_2 conversion in the UK (*M* = 0.96; *SD* = 1.00). France had the lowest rating for industrial waste application (*M* = 0.50; *SD* = 1.20), and Turkey for SC-based CO_2 conversion (*M* = 0.62; *SD* = 1.51).

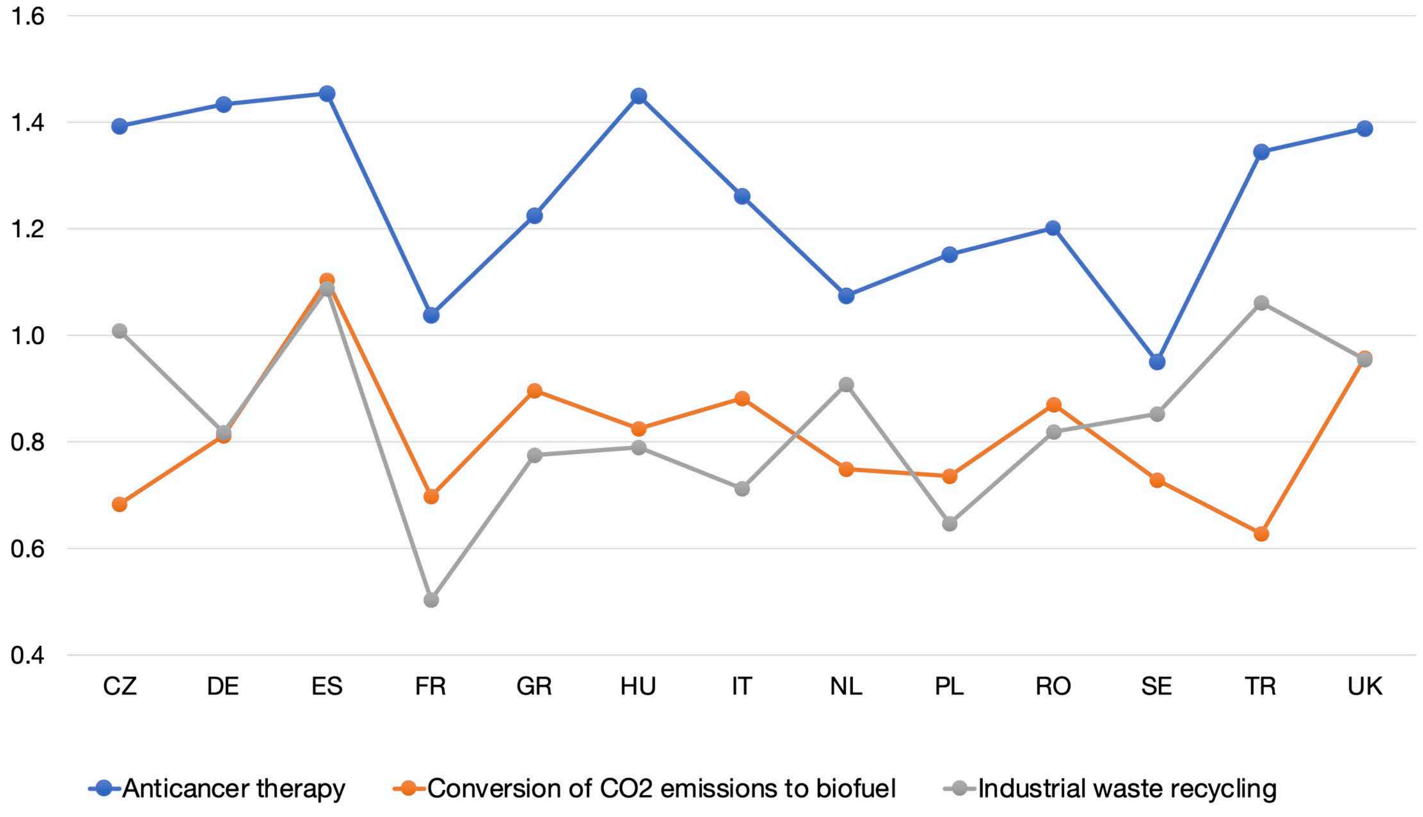

Fig 4. Synthetic cell applications acceptance per country. The x-axis presents countries: Czech Republic (CZ), Germany (DE), Spain (ES), France (FR), Greece (GR), Hungary (HU), Italy (IT), Netherlands (NL), Poland (PL), Romania (RO), Sweden (SE), Turkey (TR), United Kingdom (UK). The y-axis shows 5-point Likert scale scores recoded to numeric values: with -2 for "extremely unacceptable", -1 for "somewhat unacceptable", 0 for "neither unacceptable, nor acceptable", 1 for "somewhat acceptable", and 2 for "extremely acceptable". The reported scores varied between 0.5 and 1.45.

https://doi.org/10.1371/journal.pone.0319337.g004

Decision trees

In a DT analysis of the perceived acceptability of potential SC-based technologies, application type was identified as the root node. It split in two branches: for the two environmental applications combined, and for anticancer therapy (S9 Fig). The node for environmental applications was further split into three subgroups based on education levels. The largest of these nodes corresponded to the middle education level, with moderate acceptance score as the leading response ($M = 0.81$; $SD = 1.06$). This node further split in two branches based on gender, where male respondents provided higher acceptance scores than females ($M = 0.93$; $SD = 1.07$ vs. $M = 0.69$; $SD = 1.04$). The higher education group had the most "highly acceptable" scores ($M = 1.01$; $SD = 1.01$) and was subdivided in two nodes based on gender ($M = 1.15$; $SD = 0.99$ for males, vs. $M = 0.88$; $SD = 1.02$ for females). The lower education node had lower acceptance scores with moderate and strong acceptance prevalent but with slightly more neutral and negative answers ($M = 0.68$; $SD = 1.17$).

The node for the medical application split into three country branches. The first country node included 6 out of 13 countries (Czech Republic, Germany, Hungary, Spain, Turkey, and UK) where most respondents found SC-based therapy "highly acceptable", with the mean score of 1.41 ($SD = 0.95$). The second country node included France, Netherlands, and Sweden where SC treatment was found "somewhat acceptable" almost as often as "highly acceptable" ($M = 1.02$; $SD = 1.07$). The third country node included Greece, Italy, Poland, and Romania with scores "in between" ($M = 1.21$; $SD = 0.95$). Each of these nodes, in turn, split in 2 other branches based on education. For the first two country subsections, middle and lower education group responses were placed together, whereas for the third country node, middle and higher education groups fell under the same node. Again, higher education levels corresponded to higher acceptance scores.

In the DT for perceived willingness to use SC-based applications the data also first divided based on applications (S10 Fig). For environmental applications, the branch pattern was similar to the acceptance DT, except for the lower education subsection, which then split into three country-based groups. In Czech Republic, Romania, Spain, Turkey, and UK high acceptance was still prevalent among the respondents with lower education ($M = 0.80$; $SD = 1.20$). In Germany, Greece, and Italy it was often moderate and neutral ($M = 0.60$; $SD = 1.10$), whereas in France, Hungary, Netherlands, Poland, and Sweden it was the lowest for this population group ($M = 0.37$; $SD = 1.13$). Willingness to use SC-based therapy split in 4 branches depending on age: 45-64 years old ($M = 1.25$; $SD = 0.99$), 25-44 ($M = 1.10$; $SD = 1.04$), 18-24 ($M = 0.91$; $SD = 1.17$), and 65+ ($M = 1.37$; $SD = 0.87$). The 25–44-year-old group further split into nodes with middle and lower education ($M = 1.02$; $SD = 1.08$) and higher education ($M = 1.28$; $SD = 0.93$). Finally, the 65+ node split in two subgroups: the smallest node included respondents who did not answer the question on religious affiliation or indicated "other" ($M = 1.06$; $SD = 1.02$), whereas the main node comprised all major religions along with being non-religious ($M = 1.42$; $SD = 0.83$).

Topic modelling

The survey participants commented on their decision using open text boxes (it was not compulsory). To review key themes in a large corpus of text entries ($N = 7{,}770$) we employed topic modeling (TM). Word clouds in Figs 5–7 illustrate which words appeared most frequently in reactions to which scenario. Our TM exploration identified 8 topics for the therapy scenario, 4 for CO_2 emission conversion, and 3 for industrial waste recycling vignette. The LL/Token measure ranged between -6.63 for therapy and -6.41 for waste recycling scenario, indicating good fit. The topics with the correlated top words, topic weight and frequency within the

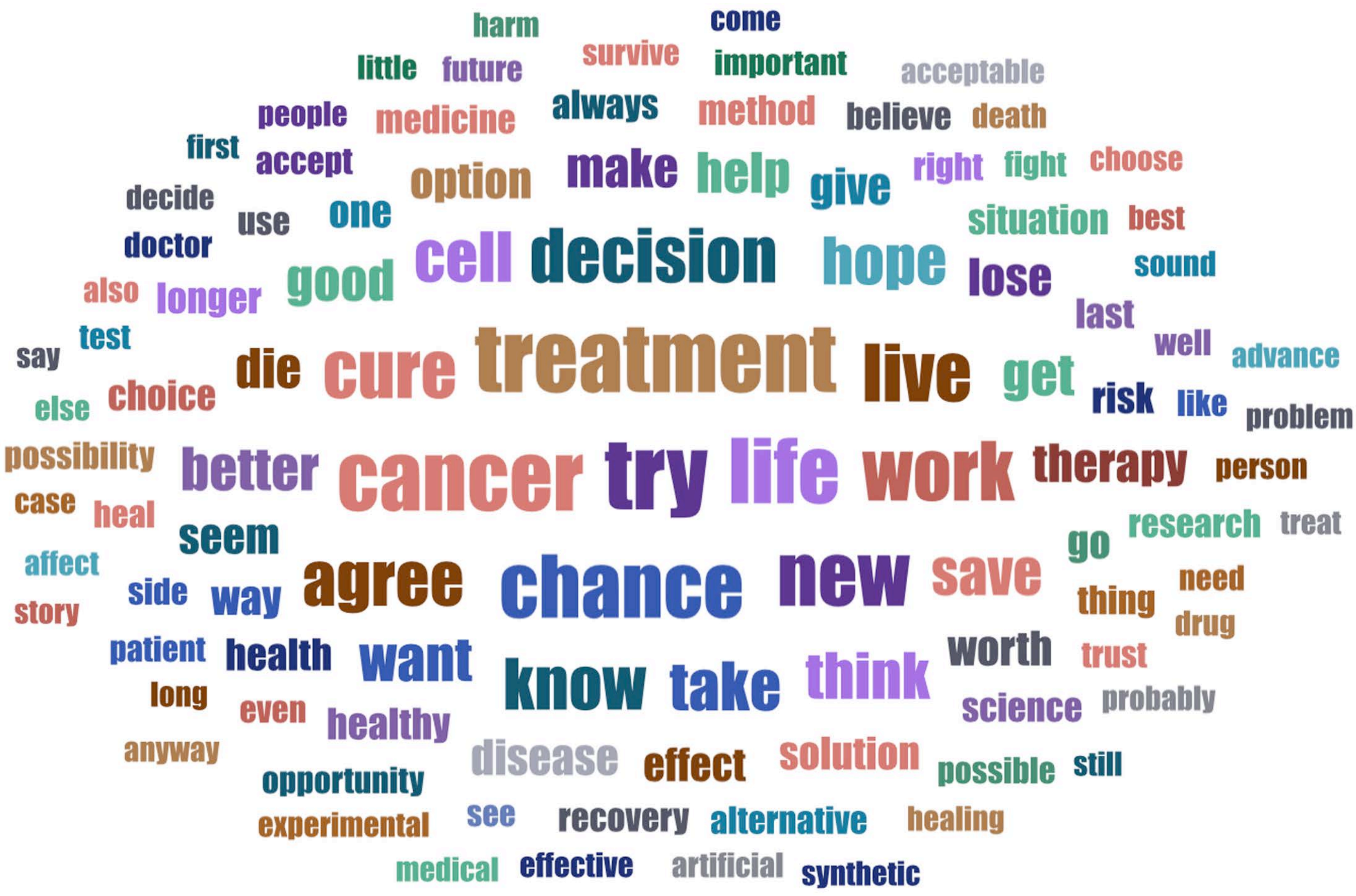

Fig 5. Word cloud: respondents' comments to scenario 1 (CS-based anticancer treatment). The figure displays words appearing at least 25 times across the reactions to the vignette (n = 2,688), with word size reflecting its frequency.

https://doi.org/10.1371/journal.pone.0319337.g005

corpus are presented in S10-12 Tables; integrated visualization of the topics with sentiment analysis elements is available in S10-12 Figs. Making sense of the topics is an interpretative process. To label and illustrate the topics, we will provide examples based on LDA tables of individual comment per topic fit (we preserved the original grammar).

Anticancer therapy. The dominant theme identified in 35% of all comments could be labeled as "a decision to use one's chance to live, a hope". Typical statements included: "many people with cancer have nothing to lose, every possible new therapeutic approach would be a ray of hope" (DE-234), "because chances are the new treatment will cure him" (CZ-048). The second topic with 18% of responses viewed SC treatment as "an alternative therapy when the old treatment is no longer effective", e.g.,: "if conventional treatment does not work, the only option is to receive a new experimental treatment or give up" (ES-139), "since the therapy taken years ago was no longer working I see no reason not to try this other alternative" (IT-040). The third topic (14% of comments) could be defined as "saving or prolonging one's life is worth it". Typical responses included: "to have a healthy life it's worth the risk, you have a chance anyway" (RO-073), "if I can regain normal living conditions, why not, life is worth living" (TR-292), "the instinct for life is the strongest human emotion" (TR-166). The fourth topic involving 10% of reactions refers to "a personal decision in hard situation", e.g.,: "the decision is up to the patient depending on what they think is best for them" (GR-050), "very

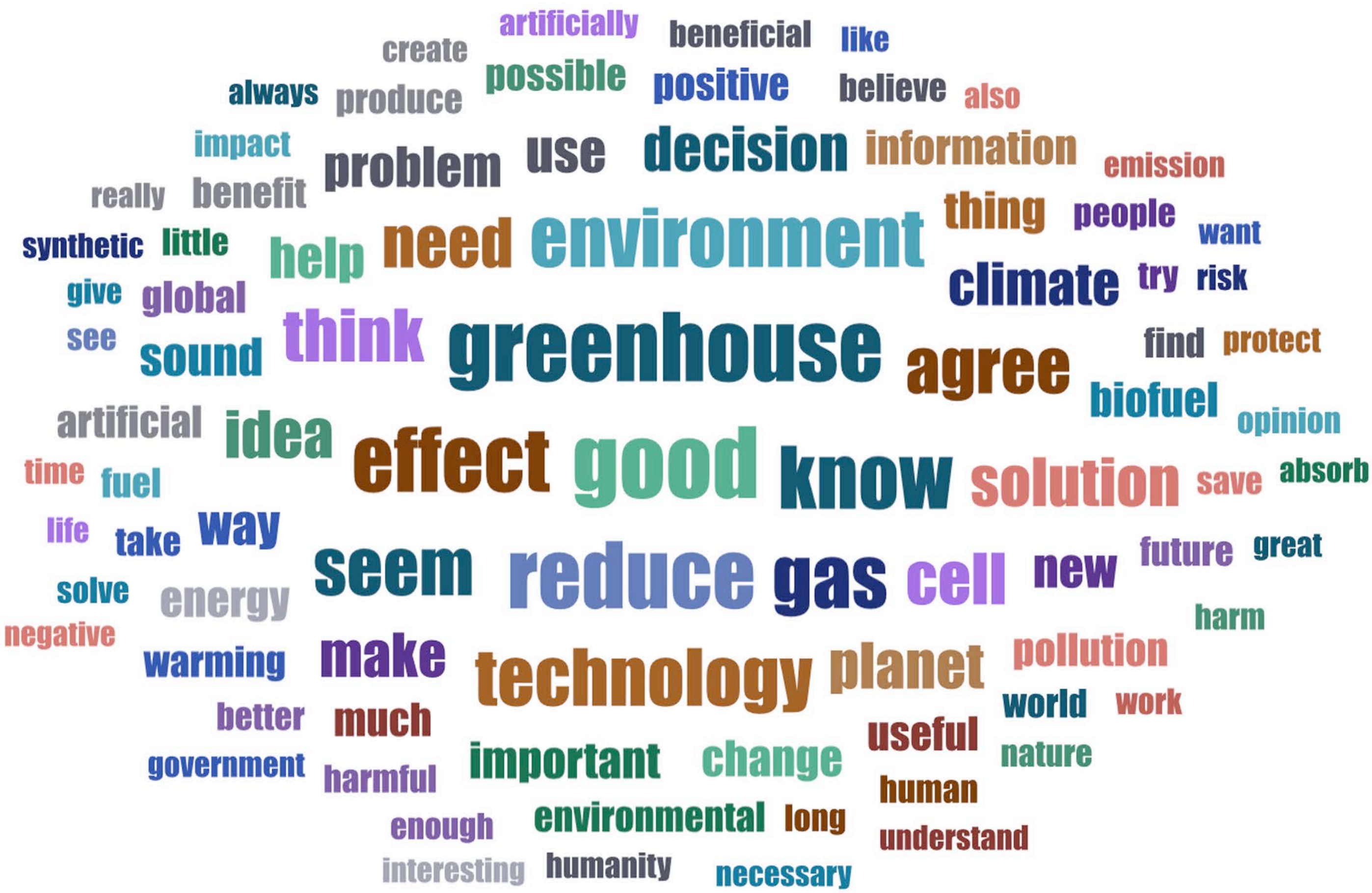

Fig 6. Word cloud: respondents' comments to scenario 2 (CS-based CO_2 emission conversion to biofuel). The figure displays words appearing at least 25 times across the reactions to the vignette (n = 2,526), with word size reflecting its frequency.

https://doi.org/10.1371/journal.pone.0319337.g006

vulnerable situation, desire to live on makes the decision reasonable" (SE-681). The fifth topic (8% of comments) mentioned potential "side effects: could artificial cells affect healthy cells?", e.g.,: "these cells can produce a drug to destroy cancer cells, but it's not clear whether there will be any side effects, or whether these artificial cells are really effective... Nothing is 100% certain" (FR-622), "the injection sounds worthwhile till you read that it 'should not' affect healthy cells. This last bit would concern me, and I would want to know more" (UK-257). The sixth topic (9% of responses) expressed one's "trust in doctors and medical research", such as the following reactions: "the development in medical science is rapid and I trust it, after all, if something new is not tried how will they discover cures..." (GR-341), "I am confident in the development of medicine and related disciplines" (HU-014). The seventh topic (5%) reflected that "information is needed on possible side effects". This topic was less stable across the algorithm runs, sometimes replaced by themes like "treatment for a life-threatening disease" or "grabbing at a straw" (such topics were not discerned, however, if a larger number of topics was chosen). Examples included: "I miss more info. Side effects, consequences, prognosis" (NL-481), "it depends how much research has gone into the new therapy and what the side effects are but I would like try it (UK-326), "I think there is too little about possible side effects" (SE-320). Finally, the eighth topic (3%) mentioned "avoiding chemotherapy": "the method seems to reduce the enormous suffering of cancer patients. It is also environmentally

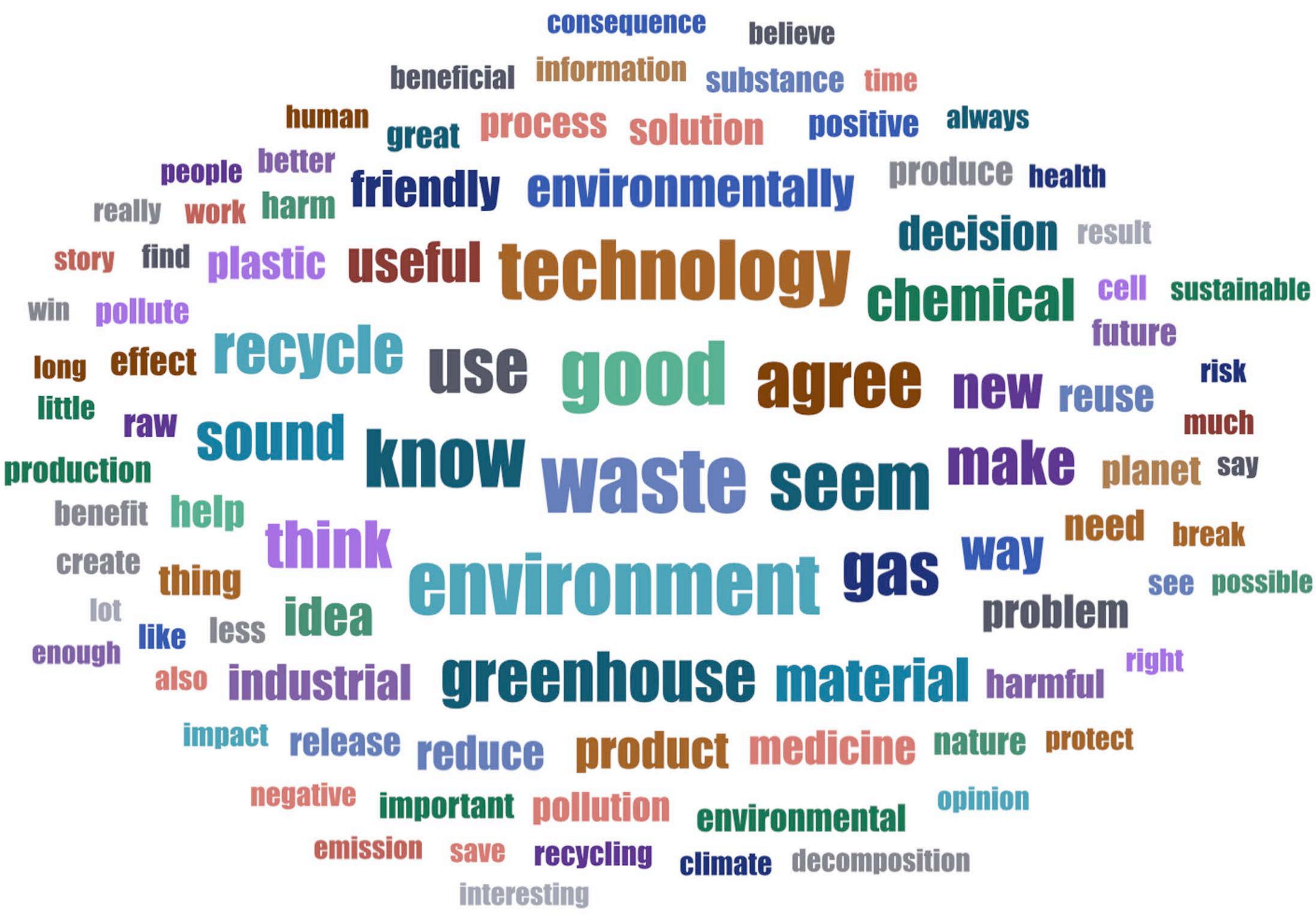

Fig 7. Word cloud: respondents' comments to scenario 3 (CS-based industrial waste recycling). The figure displays words appearing at least 25 times across the reactions to the vignette (*n* = 2,556), with word size reflecting its frequency.

https://doi.org/10.1371/journal.pone.0319337.g007

friendly compared to radiation therapy, chemotherapy, etc." (SE-141), "this way we would avoid treating the body and overloading it with chemo and radiotherapy knowing that it may not have the same effect after a long process" (ES-459).

CS-based CO_2 emission conversion to biofuel. For the SC-based application converting power plant carbon emissions to biofuel, the most prevalent topic appearing in 42% of the comments viewed it as "a good environmental idea for the planet". Typical comments within this topic were: "because it would be a solution to one of the most pressing problems facing humanity and the world" (ES-166), "even if it is experimental, they [SCs] can be the future and if it has been tested properly, we must dare to believe in the development to move forward and save the planet" (SE-513). The second topic (29%) focused on the aspect that this technology "reduces greenhouse gas while producing energy". Common responses in this theme included: "because two birds are killed with one stone, greenhouse gases are reduced and at the same time energy is gained from them" (DE-354), "this technology is environmentally friendly in two ways: it reduces the greenhouse effect and creates biofuel" (FR-022). The third topic (17% of responses) discussed that "one needs more information on synthetic cells to make decision". Some examples included: "I would want sufficient information to ensure that the process

was safe and would work in the long term" (UK-399), "we need more information on the feasibility and costs, both in financial terms and in terms of impact, because such processes seem improbable" (FR-149). The fourth topic appearing in the 14% of comments underscored "the need to solve the global warming problem". To illustrate: "because it would be a giant step forward not only in the fight against climate change, but also as a means of reversing it in a spectacular way" (ES-251), "I believe that climate disaster is a major problem and must be addressed urgently and by all means. Let alone in a constructive way" (GR-238).

Industrial waste recycling. The most common topic (53% of reactions) approached SC-based industrial waste recycling as "an environmentally friendly waste recycling technology". It included responses such as: "it is a modern method of recycling. The world is flooded with waste and its disposal is expensive and destroys the environment. This innovative technology respects the earth" (PL-471), "such technology can go a long way in helping to manage waste, which is no longer polluting the environment and is actually recycled" (HU-386). The second topic (30%) stresses that this application "turns waste into a useful material without releasing greenhouse gas". To illustrate: "the part that says no greenhouse gases are released during the process and the resulting chemical serves as a raw material for the production of resins, plastics, and medicines" (IT-495), "this way of breaking down waste is apparently not harmful to the environment and in addition, it is also very useful for humans since other products can be made from it" (NL-551). The last topic (18%), which had more variance in the top terms across runs, referred to "risk/benefit information needed on this technology". To illustrate: "I feel it is a good idea but would need more information about any risks involved from the final result" (UK-313), "it is important to make sure that this technology is safe for the environment and human health. The final decision to support this technology should be based on a comprehensive scientific, economic and social analysis, taking into account both benefits and potential risks" (PL-083).

Discussion

SynBio development holds considerable promises but also brings about technical, social, and ethical challenges. Biosafety and biosecurity are essential to prevent unintentional or deliberate damage from biotechnologies to human health and the environment. What if designed (micro)organisms escape, exchange genetic material with existing life forms or become invasive? Could they be misused? Such issues demand attention, ongoing monitoring and regulation at multiple levels [48–51]. Not surprisingly, SynBio safety (e.g., unforeseen negative consequences, potential uncontrollability, and side-effects) and, to a lesser degree, security appeared as general public concerns across studies of SynBio perceptions [23–25,29,31,52–54]. Other common concerns raised in these studies addressed socio-economic aspects of technological innovation, including equal access to its benefits, democratic governance, commercialization, and legal issues such as intellectual property rights [25,53,54]. All of these challenges would be relevant for potential SC-based applications [19,20].

Importantly, the field of SynBio application (e.g., medical vs. agri-food technologies) emerged as an essential acceptance factor [22–29], which should reflect public estimations of the balance between usefulness and potential hazards of an application. SynBio risk perception and acceptance were also associated with certain socio-demographic characteristics and worldview-related aspects, such as religion or trust in science [23,29,52,55,56]. For example, women, as well as lower educated and religious individuals were found to be more critical of SynBio [21,24,52,55,57–59].

Common public ethical concerns (sometimes converging with safety-related worries) include the unnaturalness of the new technologies, aversion to "playing God" and tampering with nature, which could cause unknown dangers [24,29–31,53,54,57]. Other considerations

may include the moral value of life [60–62], commodifying life and nature [63–65], a shift to their mechanistic understanding through the lens of assembly of biological parts [66]. SCs-based applications can evoke all of these concerns, but also bring about unique ethical challenges. Arguably, creating life-like cellular systems from bottom-up may be viewed as ontologically disturbing or incompatible with religious values [13,14,67].

This section will discuss the survey results in light of several important factors revealed by previous SynBio research: demographic aspects, application field, perceived risks and benefits, worldview-based arguments, religious values, ethical and socio-economic considerations, and ontological challenges.

The range of methodologies used in the present study helped us interpret the survey data at different levels. First, descriptive statistics approached the outcomes through the lens of demographic and cultural variables. Second, the DT algorithm identified statistically significant patterns within the data and presented them hierarchically in a predictive model. Third, topic modelling "unpacked" the motives behind participants' choices into a probabilistic model by clustering word use patterns. Finally, we quantified and analyzed the use of specific keywords to review potentially important "missing" topics.

The decisive factors: Application field and demographics

The survey responses suggest an overall positive public attitude towards SC-based medical and environmental applications, with moderate to high support and willingness to use them. As many as 80% of participants reported it was "extremely likely" or "somewhat likely" that they would have used SC-based therapy themselves under the circumstances. The willingness to authorize CS use for carbon emissions conversion to biofuel was 64% and for industrial waste recycling 66%. The proportion of respondents who found such technologies extremely or somewhat unacceptable was much lower, varying between 7% for SC therapy and 10-12% for environmental applications.

In our survey, both descriptive statistics and a data-driven DT model indicated primary importance of application field for potential support. This is in line with previous research suggesting that public reaction could be largely defined by application and its context rather than technology itself [2,23,41–43,53,68,69]. In our study, all three scenarios featured applications aimed at societal benefit and alleviation of serious global issues. Among them, the anticancer treatment scenario clearly elicited considerable support. This is consistent with multiple studies showing that therapeutic applications of novel or controversial technologies are typically among those prioritized by the publics [23–25,55,69,70]. We further speculate that strong support for SC-based anticancer therapy in our survey could be due to its combination of urgency (fighting a life-threatening disease) with an intuitive level of decision-making (one can easily relate to the situation described). Characteristically, support for the medical application increased with the respondents' age: more encounters with health issues could result in higher perceived relevance of novel therapies in one's view.

Whereas the two environmental applications received substantial support as well, 19% of participants chose a neutral position regarding the SC-based waste recycling and 22% regarding the carbon emissions conversion to biofuel. The urgency of environmental pollution and global warming issues is high, albeit may be perceived as less immediate than saving an individual's life. The uncertainty level of these applications extends to environmental impact, making mistakes potentially very costly. It may also be demanding for lay participants to evaluate the implementation of novel technologies at industry or national level. Indeed, many respondents commented they did not feel competent or confident enough to decide on such matters.

Respondents' lower education was associated with less support for potential SC applications. The same, with less prominence in the DT analysis, was true for female participants.

This is consistent with previous research [21,24,52,55]. A plausible explanation is that population groups, which believe they have little control over what happens in their communities may be more sensitive to potential hazards of novel technologies [71]. There was also some variation in SC technologies' acceptance associated with one's religion, with Protestant and non-religious respondents expressing stronger support for SC applications. However, this variation did not amount to important place in our DT model.

The DT analysis identified country clusters of stronger, weaker, and medium acceptance of the medical SC application. There were also country clusters of stronger, weaker, and medium willingness to implement the environmental SC applications, specifically among the lower educated participants. Variation in acceptability scores among the 13 countries displayed no stark regional, cultural, or economic patterns (S6-S8 Figs). Each surveyed country seemed to represent its own case, where public attitudes may have been affected by country-specific factors (institutions, companies, traditional and social media, etc.) and unique contexts. Overall, the substantial support of prospective SC applications in all participating countries reflects growing acceptance of biotechnologies in Europe [55,72].

Topics in participants' comments: Motivations for support and the need of risk assessment

As many as 92.7% of the survey participants chose to comment on their response. The topics uncovered by the LDA algorithm in these comments represent the recurrent motives for acceptance of the SC applications, as well as typical reasons behind the more cautious reactions. The therapy scenario elicited the broadest range of distinct responses. The dominant motivation centered around the importance of saving one's life ("a decision to use one's chance to live, a hope", "an alternative therapy when the old treatment is no longer effective", and "saving or prolonging one's life is worth it" amounting to 67% of reactions). In this approach, the details of a therapy appear to be secondary to its promise. Some respondents motivated the acceptability of using a novel therapy by patient's agency ("a personal choice in a hard situation", 10%) or accepted a new technology upfront ("trust in doctors and the progress of medical research", 8%). Only two topics reflect some interest in the detail of the SC treatment, namely, its safety ("side effects: could artificial cells affect healthy cells?" and "information is needed on possible side effects", 13% combined). The prevalent attitude in these two topics could be viewed as cautious rather than fearsome or prohibitive.

For the scenario of carbon emissions conversion to biofuel, the acceptance was based on the potential solution to a pressing issue of our time ("a good environmental idea for the planet" and "the need to solve the global warming problem", 56% together) and on its practical aspect ("reduces greenhouse gas while producing energy", 29%). About 17% of the respondents expressed a cautious view ("one needs more information on synthetic cells to make decision"). Again, this attitude was more judicious than inherently negative. The topics for industrial waste recycling showed similar patterns. Most comments approved the idea of "an environmentally friendly waste recycling technology" (53%) and that it "turns waste into a useful material without releasing greenhouse gas" (30%), while 18% of responses pointed at the "risk/benefit information needed on this technology" to make a balanced and prudent decision.

The perspectives uncovered via TM are consistent with the attitudes expressed by the participants in their reported scores and reflect the prevalence of strongly and moderately positive acceptance of therapeutic and environmental SC applications. The topics with more cautious approach seem to correspond mainly to neutral and perhaps also moderately negative scores. Their recurrent mentioning of need for information for balanced decision suggests that they could sometimes be qualified as conditional support. While 3-5% of participants

indicated that they found SC-based applications strongly unacceptable, their perspectives did not reach the threshold to appear as separate topics. This could be due to the lower frequency of such reactions and some participants choosing not to comment on them, but also to possible diversity within this group.

Concerns related to participants' worldview and SC ethics

Prior theoretical and empirical studies on public perceptions of SynBio discussed such considerations as "playing God", "tampering with nature", "slippery slope" and "creating" or "engineering" life, as well as ethical and social aspects, the role of religion and worldview, and the idea of "naturalness" [23,29,31,52,60,61]. These themes remained beyond our algorithm-defined topics. We will now zoom in at these questions within our survey.

Generic biotechnology concerns: Clash with religious worldviews, interfering with nature, and naturalness vs. artificiality. Religiousness is often associated with a more critical view of SynBio [24,29,52]. At the same time, the well-known argument of "playing God" – although it may have teleological "natural law" roots [73] – is not raised by theologians [60,61] and is more likely to be used by "secular critics of the field" [74, p. 153]. The argument of "playing God" aligns with the aversion to "tampering with nature", and "slippery slope" themes. Metaphorically expressed, these reactions often include intrinsic concerns such as human fallibility and unintended ethical, social or safety consequences [31,53]. Unease about interfering with nature could overlap with a preference for naturalness [29], and the latter represents a distinct attitude, where "natural" is heuristically associated with benevolence, safety and morality, as opposed to artificial products [75,76]. This attitude might be stronger in Western countries where people may feel more alienated from nature [33,75]. To evaluate, to what extent such worldview-related concerns appeared in our survey, we examined all comments containing specific words and word combinations.

Only 7 individuals mentioned God, religion or confession in their comments (words like "divine", "sacred", "lord", etc. did not appear in the sample), so we can list them here: "cancer tumor is created by God so man should NOT intervene" (NL-599); "I prefer to leave my life in God's hands and let him decide. I don't think that's acceptable" (RO-199); "if hope means life, that hope is Allah's command" (TR-284); "Islam religion" (TR-364), "I am a person of faith, and this is not decent for a Christian" (PL-674); "we will not replace God" (PL-542); "for the creation of artificial life. It seems to me to play gods" (ES-448). Most of these comments discuss whether to undergo a treatment, and the last three react to the waste recycling technology. The last comment belongs more to the "interference with nature" and "creating life" themes. We can conclude that in our survey religious worldview as such was not a reason for serious concerns about SCs.

We reviewed all text entries containing the word "nature" (excluding semantically different usage, such as "the nature of question"), and lemmatized versions of words "interfere", "play", "temper", "mess", "manipulate", "slippery", "Frankenstein", "creature", monster". There were in total 31 comments associated with the theme of "interfering with nature" or being against the nature. Some examples are: "because it is an interference with nature" (PL-483), "let's stop trying to change nature" (FR-377), "this is against nature (DE-480)", or "man should not interfere with such matters, greenhouse gases were, are and will be, playing with nature is not a good idea, everything evolves including the earth which adapts to conditions as they are" (PL-414). Such comments appeared in 0.4% of all responses, sometimes overlapping with the theme of "artificial vs. natural".

For "artificial" vs. "natural" motif, we reviewed all text entries with the words "(un) natural(ness)" (excluding semantically irrelevant use, such as "it's natural to want to survive" or "natural resources"), "(non-)organic", "artificial(ly/ity)" and "synthetic(ally)". There were

in total 268 such text entries (3.4% of responses), and part of them mentioned "artificial" or "synthetic" in positive or neutral context, such as "artificially produced cells will contribute to reducing the greenhouse effect" (PL-240), "the benefit to society as a whole is great. Since the cells are artificially produced, no one is harmed" (DE-137), "because it enables the cancer to be defeated by the synthetic cells" (DE-245), "this technology reduces waste with no apparent environmental consequences. I do not believe there is anything intrinsically wrong with synthetically producing cells" (UK-662). If we exclude such entries and correct for several terms occurring within a single comment, we will have 165 comments (2.1% of all responses) involving "artificial vs. natural" argument. To illustrate: "I am a believer in natural remedies and self-healing" (HU-236), "I don't like artificial things, even if its greenhouse, they should remain natural (TR-649), "I favor natural solutions over artificial ones" (NL-027), "I'm for something natural not synthetic" (RO-108), "interesting but not natural and therefore dangerous" (FR-643), "it's a man made process, rather than a natural process. It continues the life of plastics which I would rather not have at all" (UK-526).

Whereas our estimations may not be precise (e.g., larger vocabularies could be used, with refined theme analysis per comment), they provide a useful indication of the presence and frequency of these "hidden" topics. Worldview-related arguments were thus involved in ca. 2.5% of all responses. Overall, although we should take these considerations seriously, they do not refer to features that are unique for SCs. Rather, they could be applied to SynBio or biotechnology in general.

Ethical challenges specific for SCs. Since cells constitute the basic unit of life, the question emerges whether the building of (partly) functional cells in a laboratory counts as creation of life, and if so, would this have moral implications? The top-down SC (a bacterial cell with self-replicating synthetic genome) engineered by Craig Venter's center in 2010 was often discussed in the media as creation of artificial life, partly prompted by Venter's promotion of this advance [77]. The US presidential bioethics commission assessing the ethics of this new technology concluded that it did "not amount to creating life as either a scientific or a moral matter" as it relied on an existing host and did "not represent the creation of life from inorganic chemicals alone" [74, p. 3]. Bottom-up SCs, involving emergent properties [78] may bring about ethical concerns about not just re-engineering life but assembling it [13]. In the future, such technologies could shatter the "aura" of life as we know it [14] and make us rethink some fundamental categories and practices [20]. Scientists working on bottom-up SCs currently consider them not alive, "not quite alive", or as a "proxy for life" [6, p. 162, 166; 79], the main criteria of "aliveness" being autonomous self-replication and life-like behavior [79]. A range of approaches to assessing the "life-likeness" of SCs (such as Turing test) is currently under discussion [80,81]. While construction of living cells ("Life 2.0") remains a remote goal in the field [80], non-living SCs carry advantages for biotechnological applications [6].

What we consider life is relevant for scientific research and risk prevention, but also has ethical and normative implications and should allow societal discussion [82]. As we prepare for closer engagement with the societal stakeholders, we want to know, whether our survey participants – not primed by leading questions or comparisons – associated human-made, synthetic cells with creating life, challenging conventional ideas about it, or crossing some moral threshold. Which ethical considerations did they express regarding the SCs and SC-based technologies? To answer these questions, we reviewed all comments, which mentioned creation of life or ethics.

To assess the "creating/engineering life" argument we searched for the following words and lemmatized word combinations with semantically relevant use: organism, lab(oratory), (non-)living, (in)organic, biological, life + artificial/synthetic/engineer/create. Out of 27 comments, only 6 were closely related to this discussion: "artificial cell structures have been

developed using inorganic substances” (RO-662), “the creation of new living cells carries the risk of a lack of control over their reproduction and impact on human health” (PL-286), “the fact that the government approves does not mean that it is ethically justified. Reducing the greenhouse effect is good. The only question is how far one should go in the sense of how engineerable life is” (NL-112), “why pack artificial cells???? in not long they will create an artificial human” (PL-144), “for the creation of artificial life. It seems to me to play gods” (ES-448), “the technology described seems to allow the environment and hazardous waste to be easily transformed into something useful. It sounds like something that industry needs. I’m not sure what ‘synthetic cells’ can be (problems with translation?) but if it means organisms made in the lab, which can be no danger in themselves, then the decision sounds right” (SE-501). Other relevant comments in this group mentioned risk and benefit factors (thrice), the importance of the context in which such engineered cells were used (twice), a moral dilemma of using cells (once), genetically engineered cells (once), need for more information on artificially engineered cells (once), plainly disagreed with artificially engineered cells (once) or said such application was not credible (once). The life status of SCs was thus discussed extremely rarely.

Ethical considerations (lemmatized words: ethics, moral) were mentioned in 40 comments. Only 9 respondents found the use of SCs unethical or were not sure about it, with comments such as “ethics” (RO-222), “I don’t know if it’s ethical and ecological” (FR-147), “lack of ethics” (PL-718), “it’s not ethical” (TR-331), “technology seems useful but I don’t know if it’s ethical” (PL-098). While 18 respondents expressed a view that the use of SCs was ethical, another 8 found that one needs to know/check if it was ethical, and 5 individuals said that the benefits of such applications were more important than ethics. Compared to other cell technologies (such as stem cells or CRISPR) SCs seemed to avoid moral criticism of cell use.

Societal aspects. To evaluate the discussion on socio-economic, political and legal aspects of SC-based applications, we reviewed the comments with the following words (lemmatized to allow different forms): society, social, government, political, policy, nation, economy, cost, profit, expense, budget, finance, labor, worker, job, factory, company, business, corporation, private, rich, elite, corrupt, equal, distribute, justice, law, legal. The words related to social justice and distributive equality (equal, distribute, justice, private, elite) did not appear in the corpus at all. Other words appeared in 151 comments (1.9% of all text entries), typically in combination with other topics and often mentioning societal or economic worth of SC technologies.

Overall, in our survey, socio-economic perspectives did not amount to distinct themes. One of the reasons may be the brevity and exploratory character of the study. In this set-up, the beneficial functions of SC-based applications and their potential risks likely appeared as a more salient aspect of the question. The results could have been different if the survey had studied not only the primary attitudes but also had involved more deliberate and effortful processing: e.g., by directly asking questions on potential societal issues with these new technologies. In general, less focus of our respondents on ethical and social issues vs. risks and benefits is in line with prior quantitative research. For example, in the Eurobarometer 2010 survey, SynBio social and ethical issues were selected least frequently compared to risks/benefits as a topic to learn about for a hypothetical referendum vote [59].

Pragmatics and ethics

Our vignettes did not explicitly mention designing (pseudo)organisms or working with genes. In this context, we detected no public distrust towards SCs or related technologies. Our scenarios did stress the artificial origin of the cells used. The sheer idea of human-made cells doing work for public health and environment was welcomed by most respondents, apparently

without clashing with religious worldviews or eliciting associations with “creating life”. It is likely that cells are not generally associated with “life” as a moral category, as long as they are not human, embryonic, or chimeric. Studies on SynBio perceptions suggest that evolutionary distance of a species from humans may affect the acceptance of its genetic modification [29,33]. In an anthropocentric perspective, constructing microorganisms does not attain the moral significance of creating life [60,61]. But it is also plausible that many people implicitly view cells or microorganisms as non-living, if they do not exist independently of their human-programmed tasks, much in the same way, as SC scientists find current SCs “not quite alive”.

Most public concerns about SCs, which appeared in 13–18% of survey responses, involved practical considerations about control, risk/benefit balance, remote consequences, and the need for thorough information. Concerns about the naturalness of SC technologies were raised in ca. 2.1% of comments. These types of criticism and unease are not unique to SCs but common to biotechnology in general. It seems that SCs and technologies discussed in the study elicited no new critical arguments or ethical concerns specific to SCs. Ethics as such was not much disputed by the survey participants, but we may argue that the study shows implicit ethical approval of SCs in the service of medicine and sustainability.

Whereas new societal, ethical, and philosophical issues regarding SCs will likely arise in the future studies (especially, qualitative ones, such as focus groups), the preliminary responses to the prospect of artificially designed cells may reflect two important points. First, they suggest that, for most participants, the idea of SCs did not (yet) cross the threshold of ontologically radical or ethically disturbing. Second, they point at the prevalence of a practical problem-solving attitude when it comes to serious healthcare and environmental challenges. Importantly, this attitude is not indifferent to ethics or ethics-independent but is grounded in values such as health, environmental protection and sustainability.

Limitations and future directions

The main limitation of the current survey is that it could only reflect some preliminary attitudes to SCs. Our study was limited by its cross-sectional character (collecting public responses at a single moment in time), geographical region (Europe), and the hypothetical nature of scenarios (which is a necessity for anticipatory approach). The surveyed countries were mostly high income, except for Romania and Turkey, which belong to the upper middle-income group. The survey involved no socio-economic or worldview measures such as income, or political preferences.

A one-time attitude measurement suggests limited predictive validity. However, as conceptualized in the dual processing models and supported by empirical evidence, low processing time is a moderator of higher validity in measuring attitudes [83,84]. In our study, the brevity of the survey helped elicit more automatic reactions in respondents, which is relevant for assessing attitudes. To obtain such reactions we avoided framing participants by emotionally colored words (either positive or negative), comparisons, or leading questions. Not providing detailed information was, among the rest, intended to keep the participants open to their own understanding of the matter, guided by their own values and immediate considerations. However, no story could (or should) be entirely neutral. Discussing societally important topics and referring to positive decisions made in the scenarios may inadvertently have involved some kind of priming. Therefore, an obvious study limitation is that alternative vignette details such as choosing a less known or dangerous disease, or providing more information on how SCs are produced, would likely affect the respondents' reactions. Such a constraint was difficult to avoid in exploratory survey. Insights from the current study, adjusted for ongoing SC and SynBio advances, could help develop more detailed questionnaires or contrastive quasi-experimental vignettes focusing on specific societal and ethical issues.

Other study limitations involved more technical challenges. For example, in a multilingual study with automated translations of the participants' comments (even though unclear passages were reviewed with the native speakers) some comments could lose their nuances. With topic modeling, smaller yet distinct topics (including some critical voices) might have not been extracted by an ML algorithm. Recognizing these limitations, we assume that using several methods jointly, including data-driven techniques, helped us achieve a multidimensional view of participants' reactions.

Future research should explore how the publics evaluate different characteristics of SCs and the prospects of related technologies in multiple fields. Qualitative studies such as focus groups should engage various types of societal stakeholders and promote public discussion on opportunities and challenges, as well as philosophical and policy implications related to SCs. We see this first exploratory survey of public attitudes to potential SC applications as a pre-consultation of publics and a step towards consulting the society in a more in-depth way.

Conclusion

Potential technologies involving SCs may be used to advance medicine and address environmental challenges such as global warming. They could become part of the future economy and deeply affect our relationship with nature, including the way we view life and where we place the border between the living and non-living. We conducted a large European survey with nationally representative population samples, focusing on societally relevant areas of SC technologies application. Using vignettes allowed us to place our questions in practical context increasing ecological validity and to assess the participants' attitudes through the lens of decision-making. The discussion of these human-made cells did not elicit worries about "creating life" or ethical concern specific for SCs. The participants' reactions seemed to have been guided mainly by their perceived benefit and risk estimations of the SC applications. Our findings suggest considerable potential support for SC-based technologies in healthcare and sustainability fields across Europe. Whether potential support would become informed and active depends, among the rest, on further public engagement and transparent democratic governance of such technologies.

Supporting information

S1 File. S1 Table. Demographics/Population characteristics. Abbreviations here and below: Czech Republic (CZ), Germany (DE), Spain (ES), France (FR), Greece (GR), Hungary (HU), Italy (IT), Netherlands (NL), Poland (PL), Romania (RO), Sweden (SE), Turkey (TR), United Kingdom (UK). S2 Table. Criteria for excluding survey data from statistical and/or textual analysis. S3 Table. Sample size per country before and after removing invalid entries. S4 Table. Mean acceptability of SC-based applications. Anticancer therapy (V1), Conversion of CO2 emissions to biofuel (V2) and Industrial waste recycling (V3). S5 Table. Synthetic cell applications acceptance per gender: Mean and Standard Deviation. Synthetic cell applications acceptance per gender: Mean and Standard Deviation. S6 Table. Synthetic cell applications acceptance per age category: Mean and Standard Deviation. S7 Table. Synthetic cell applications acceptance per education level: Mean and Standard Deviation. S8 Table. Synthetic cell applications acceptance per religion: Mean and Standard Deviation. S9 Table. Synthetic cell applications acceptance per country: Mean and Standard Deviation. S10 Table. Topic modeling of the respondents' comments to scenario 1 (anticancer therapy). The "topic" column contains a "label" description reflecting the author's interpretation of the topic as based on the top words and confirmed by typical entries per topic. Weight indicates relative prominence of the topics within the model (numeric values depend on the number of iterations). LL/T

(log-likelihood per token) represents the overall model fit. S11 Table. Topic modeling of the respondents' comments to scenario 2 (conversion of CO_2 emissions to biofuel). The "topic" column contains a description reflecting the author's interpretation of the topic as based on the top words and confirmed by typical entries per topic. Weight indicates relative prominence of the topics within the model (numeric values depend on the number of iterations). LL/T (log-likelihood per token) represents the overall model fit. S12 Table. Topic modeling of the respondents' comments to scenario 3 (industrial waste recycling). The "topic" column contains a description reflecting the author's interpretation of the topic as based on the top words and confirmed by typical entries per topic. Weight indicates relative prominence of the topics within the model (numeric values depend on the number of iterations). LL/T (log-likelihood per token) represents the overall model fit. S1 Fig. Mean acceptability of SC-based applications. Anticancer therapy (V1), Conversion of CO_2 emissions to biofuel (V2), and Industrial waste recycling (V3). S2 Fig. Perceived acceptability of SC-based applications per gender. S3 Fig. Perceived acceptability of SC-based applications per age. S4 Fig. Perceived acceptability of SC-based applications per education level. S5 Fig. Perceived acceptability of SC-based applications per religion. S6 Fig. Perceived acceptability of SC-based anticancer therapy (scenario 1) per country. S7 Fig. Perceived acceptability of SC-based conversion of CO_2 emissions to biofuel (scenario 2) per country. S8 Fig. Perceived acceptability of SC-based industrial waste recycling (scenario 3) per country. S9 Fig. Perceived acceptability of SC-based applications: Decision Tree. Bars in the boxes show Likert scale score distribution. S10 Fig. Perceived willingness to use SC-based applications: Decision Tree. Bars in the boxes show Likert scale score distribution. S11 Fig. Topic modeling process overview. S12 Fig. Visualization of integrated TM and SA: SC anticancer therapy scenario. The centrality of the box and the intensity of yellow reflect topic weight (prominence) vs. other topics. Word size represents relative frequency of the words within the topic. Additionally, word colors indicate sentiment: green for positive, red for negative, and blue for neutral. S13 Fig. Visualization of integrated TM and SA: SC-based conversion of CO_2 emissions to biofuel. The centrality of the box and the intensity of yellow reflect topic weight (prominence) vs. other topics. Word size represents relative frequency of the words within the topic. Additionally, word colors indicate sentiment: green for positive, red for negative, and blue for neutral. S14 Fig. Visualization of integrated TM and SA: CS-based industrial waste recycling scenario. The centrality of the box and the intensity of yellow reflect topic weight (prominence) vs. other topics. Word size represents relative frequency of the words within the topic. Additionally, word colors indicate sentiment: green for positive, red for negative, and blue for neutral.
(PDF)

Acknowledgements

The authors thank all colleagues and friends who helped us cross-validate the vignettes and translate the questionnaire.

Author contributions

Conceptualization: Olga Rook, Hub Zwart, Marileen Dogterom.

Data curation: Olga Rook.

Formal analysis: Olga Rook.

Funding acquisition: Hub Zwart, Marileen Dogterom.

Investigation: Olga Rook.

Methodology: Olga Rook, Hub Zwart.

Project administration: Olga Rook.

Supervision: Hub Zwart, Marileen Dogterom.

Visualization: Olga Rook.

Writing – original draft: Olga Rook.

Writing – review & editing: Olga Rook, Hub Zwart.

References

1. Polizzi KM. What is synthetic biology? Humana Press; 2013. https://doi.org/10.1007/978-1-62703-625-2_1
2. Benner SA, Sismour AM. Synthetic biology. Nat Rev Genet. 2005;6(7):533–43. https://doi.org/10.1038/nrg1637 PMID: 15995697
3. Deplazes A. Piecing together a puzzle: an exposition of synthetic biology. EMBO Rep. 2009;10(5):428–32. https://doi.org/10.1038/embor.2009.76 PMID: 19415076
4. Garner KL. Principles of synthetic biology. Essays Biochem. 2021;65(5):791–811. https://doi.org/10.1042/EBC20200059 PMID: 34693448
5. Chopra P, Kamma A. Engineering life through synthetic biology. In Silico Biol. 2006;6(5):401–10. https://doi.org/10.3233/ISB-00253 PMID: 17274769
6. Adamala KP, Dogterom M, Elani Y, Schwille P, Takinoue M, Tang TD. Present and future of synthetic cell development. Nat Rev Mol Cell Biol. 2004;25(3):162–7. https://doi.org/10.1038/s41580-023-00686-9 PMID: 38102450
7. Kumar N, Samant S, Singh K, Reshamwala SM. Minimal cells and genome minimization: top-down and bottom-up approaches to construct synthetic cells. In: Biomanufacturing for sustainable production of biomolecules. Singapore: Springer Nature Singapore; 2023. p. 17–44. https://doi.org/10.1007/978-981-19-7911-8_2
8. Luo Z, Yang Q, Geng B, Jiang S, Yang S, Li X, et al. Whole genome engineering by synthesis. Sci China Life Sci. 2018;61(12):1515–27. https://doi.org/10.1007/s11427-018-9403-y PMID: 30465231
9. Gibson DG, Glass JI, Lartigue C, Noskov VN, Chuang RY, Algire MA, et al. Creation of a bacterial cell controlled by a chemically synthesized genome. Science. 2010;329(5987):52–6. https://doi.org/10.1126/science.1190719 PMID: 20488990
10. Göpfrich K, Platzman I, Spatz JP. Mastering complexity: towards bottom-up construction of multi-functional eukaryotic synthetic cells. Trends Biotechnol. 2018;36(9):938–51. https://doi.org/10.1016/j.tibtech.2018.03.008 PMID: 29685820
11. Schwille P. How simple could life be? Angew Chem Int Ed Engl. 2017;56(37):10998–1002. https://doi.org/10.1002/anie.201700665 PMID: 28678351
12. Bedau MA, Parke EC. Social and ethical issues concerning protocells. In: Protocells: bridging nonliving and living matter. Cambridge, Mass.: MIT Press; 2008. p. 641–53. https://doi.org/10.7551/mitpress/7590.003.0035
13. Bedau MA, Parke EC, Tangen U, Hantsche-Tangen B. Social and ethical checkpoints for bottom-up synthetic biology, or protocells. Syst Synth Biol. 2009;3(1-4):65–75. https://doi.org/10.1007/s11693-009-9039-2 PMID: 19816801
14. Broeks D, Hendlin Y, Zwart H. Fake cells and the aura of life: a philosophical diagnostic of synthetic life. Endeavour. 2022;46(4):100845. https://doi.org/10.1016/j.endeavour.2022.100845 PMID: 36194916
15. Sato W, Zajkowski T, Moser F, Adamala KP. Synthetic cells in biomedical applications. Wiley Interdiscip Rev Nanomed Nanobiotechnol. 2022;14(2):e1761. https://doi.org/10.1002/wnan.1761 PMID: 34725945
16. Miller TE, Beneyton T, Schwander T, Diehl C, Girault M, McLean R, et al. Light-powered CO2 fixation in a chloroplast mimic with natural and synthetic parts. Science. 2020;368(6491):649–54. https://doi.org/10.1126/science.aaz6802
17. Lussier F, Staufer O, Platzman I, Spatz JP. Can bottom-up synthetic biology generate advanced drug-delivery systems? Trends Biotechnol. 2021;39(5):445–59. https://doi.org/10.1016/j.tibtech.2020.08.002 PMID: 32912650
18. Tõnurist P, Hanson A. OECD Working Papers on Public Governance No. 44. OECD Working Papers on Public Governance. 2020(44):1–46.

19. Aarts N, Bovenberg R, Dogterom M, van Est R, Gerritsen J, Macnaghten P, et al. Society and synthetic cells: a position paper by the Future Panel on Synthetic Life. Rathenau Instituut; 2022.

20. Habets MG, Zwart HA, van Est R. Why the synthetic cell needs democratic governance. Trends Biotechnol. 2021;39(6):539–41. https://doi.org/10.1016/j.tibtech.2020.11.006

21. Jin S, Clark B, Kuznesof S, Lin X, Frewer LJ. Synthetic biology applied in the agrifood sector: public perceptions, attitudes and implications for future studies. Trends Food Sci Technol. 2019;91:454–66. https://doi.org/10.1016/j.tifs.2019.07.025

22. Pauwels E, Lovell A, Rouge E. Trends in American and European press coverage of synthetic biology–tracking the years 2008-2011. Synthetic Biology Project; 2012. Washington, D.C.: Woodrow Wilson International Center for Scholars. Available from: https://www.wilsoncenter.org/publication/trends-american-and-european-press-coverage-synthetic-biology-2008-2011

23. Pauwels E. Public understanding of synthetic biology. BioScience. 2013;63(2):79–89. https://doi.org/10.1525/bio.2013.63.2.4

24. Hart Research Associates. Awareness and impressions of synthetic biology: a report of findings based on a national survey among adults. Synthetic Biology Project, The Woodrow Wilson International Center For Scholars; 2013. Available from: https://www.wilsoncenter.org/publication/awareness-impressions-synthetic-biology

25. Starkbaum J, Braun M, Dabrock P. The synthetic biology puzzle: a qualitative study on public reflections towards a governance framework. Syst Synth Biol. 2015;9:147–57. https://doi.org/10.1007/s11693-015-9182-x

26. Ineichen C, Biller-Andorno N, Deplazes-Zemp A. Image of synthetic biology and nanotechnology: a survey among university students. Front Genet. 2017;8:122. https://doi.org/10.3389/fgene.2017.00122 PMID: 28979291

27. Borgers M. Representation of synthetic biology in Dutch newspapers, M.Sc. Thesis; 2017. Available from: https://studenttheses.uu.nl/handle/20.500.12932/25816

28. Kemal RA. Perception of synthetic biology application for biodiversity conservation among life science students in Institut Teknologi Bandung, Indonesia. Nusant Biosci. 2018;10(1):36–40. https://doi.org/10.13057/nusbiosci/n100105

29. Dragojlovic N, Einsiedel E. Framing synthetic biology: evolutionary distance, conceptions of nature, and the unnaturalness objection. Sci Commun. 2013;35(5):547–71. https://doi.org/10.1177/1075547012470707

30. Pauwels E. Review of quantitative and qualitative studies on US public perceptions of synthetic biology. Syst Synth Biol. 2009;3(1-4):37–46. https://doi.org/10.1007/s11693-009-9035-6 PMID: 19816798

31. Carter L, Mankad A, Hobman EV, Porter NB. Playing God and tampering with nature: popular labels for real concerns in synthetic biology. Transgenic Res. 2021;30(2):155–67. https://doi.org/10.1007/s11248-021-00233-2 PMID: 33502671

32. Hobman EV, Mankad A, Carter L. Public perceptions of synthetic biology solutions for environmental problems. Front Environ Sci. 2022;10:928732. https://doi.org/10.3389/fenvs.2022.928732

33. Jin S, Dawson IG, Clark B, Li W, Frewer LJ. Chinese public perceptions of food applications based on synthetic biology. Food Qual Prefer. 2023;110(3):104950. https://doi.org/10.1016/j.foodqual.2023.104950

34. Krinsky N, Kaduri M, Zinger A, Shainsky-Roitman J, Goldfeder M, Benhar I, et al. Synthetic cells synthesize therapeutic proteins inside tumors. Adv Healthc Mater. 2018;7(9):1701163. https://doi.org/10.1002/adhm.201701163

35. Almqvist H, Veras H, Li K, Garcia Hidalgo J, Hulteberg C, Gorwa-Grauslund M, et al. Muconic acid production using engineered Pseudomonas putida KT2440 and a guaiacol-rich fraction derived from kraft lignin. ACS Sustain Chem Eng. 2021;9(24):8097–106. https://doi.org/10.1021/acssuschemeng.1c00933

36. Diesner J. ConText: software for the integrated analysis of text data and network data. Social and semantic networks in communication research; 2014. Available from: https://context.ischool.illinois.edu

37. Diesner J, Aleyasen A, Chin C, Mishra S, Soltani K, Tao L, et al. ConText: network construction from texts [Software]; 2020. Available from: https://context.ischool.illinois.edu

38. McCallum AK. Mallet: a machine learning for languagetoolkit; 2002. Available from: https://mimno.github.io/Mallet

39. Wilson T. Recognizing contextual polarity in phrase-level sentiment analysis. In: Proceedings of HLT/EMNLP; 2005.

40. Khosrowabadi N, Hoberg K, Imdahl C. Evaluating human behaviour in response to AI recommendations for judgemental forecasting. Eur J Oper Res. 2022;303(3):1151–67. https://doi.org/10.1016/j.ejor.2022.03.017
41. Varian HR. Big data: New tricks for econometrics. J Econ Perspect. 2014;28(2):3–28. https://doi.org/10.1257/jep.28.2.3
42. Shmueli G, Yahav I. The forest or the trees? Tackling Simpson's paradox with classification trees. Prod Oper Manage. 2018;27(4):696–716. https://doi.org/10.1111/poms.12819
43. Lantz B. Machine learning with R: expert techniques for predictive modeling. Packt Publishing Ltd; 2019.
44. Yang Y, Yi F, Deng C, Sun G. Performance analysis of the CHAID algorithm for accuracy. Mathematics. 2023;11(11):2558. https://doi.org/10.3390/math11112558
45. Alghamdi R, Alfalqi K. A survey of topic modeling in text mining. Int. J. Adv. Comput. Sci. Appl. (IJACSA). 2015;6(1).
46. Blei DM. Probabilistic topic models. Commun ACM. 2012;55(4):77–84. https://doi.org/10.1145/2133806.2133826
47. Albalawi R, Yeap TH, Benyoucef M. Using topic modeling methods for short-text data: a comparative analysis. Front Artif Intell. 2020;3:42. https://doi.org/10.3389/frai.2020.00042 PMID: 33733159
48. Trump BD, Florin MV, Perkins E, Linkov I, editors. Emerging threats of synthetic biology and biotechnology: addressing security and resilience issues [Internet]. Dordrecht (DE): Springer; 2021. https://doi.org/10.1007/978-94-024-2086-9 PMID: 36121971
49. Wang F, Zhang W. Synthetic biology: recent progress, biosafety and biosecurity concerns, and possible solutions. J Biosaf Biosecur. 2019;1(1):22–30. https://doi.org/10.1016/j.jobb.2018.12.003
50. Acevedo-Rocha CG. The synthetic nature of biology. In: Ambivalences of creating life: societal and philosophical dimensions of synthetic biology; 2016. p. 9–53. https://doi.org/10.1007/978-3-319-21088-9_2
51. Braun M, Fernau S, Dabrock P. (Re-) designing nature? An overview and outlook on the ethical and societal challenges in synthetic biology. Adv Biosyst. 2019;3(6):1800326. https://doi.org/10.1002/adbi.201800326
52. Akin H, Rose KM, Scheufele DA, Simis-Wilkinson M, Brossard D, Xenos MA, et al. Mapping the landscape of public attitudes on synthetic biology. BioScience. 2017;67(3):290–300. https://doi.org/10.1093/biosci/biw171
53. Betten AW, Broerse JEW, Kupper F. Dynamics of problem setting and framing in citizen discussions on synthetic biology. Public Underst Sci. 2018;27(3):294–309. https://doi.org/10.1177/0963662517712207 PMID: 28597721
54. Steurer W. "Some Kind of Genetic Engineering… Only One Step Further"—Public Perceptions of Synthetic Biology in Austria. In: Ambivalences of creating life: societal and philosophical dimensions of synthetic biology. Cham: Springer International Publishing. 2015. p. 115–40. https://doi.org/10.1007/978-3-319-21088-9_6
55. Eurobarometer Special. European citizens' knowledge and attitudes towards science and technology. Eurobarometer; 2021. Available from: https://doi.org/10.2775/303708
56. Većkalov B, van Stekelenburg A, van Harreveld F, Rutjens BT. Who is skeptical about scientific innovation? Examining worldview predictors of artificial intelligence, nanotechnology, and human gene editing attitudes. Sci Commun. 2023;45(3):337–66. https://doi.org/10.1177/10755470231184203
57. Dragojlovic N, Einsiedel E. Playing god or just unnatural? Religious beliefs and approval of synthetic biology. Public Underst Sci (Bristol, England). 2013;22(7):869–85. https://doi.org/10.1177/0963662512445011 PMID: 23825242
58. Braman D, Mandel GN, Kahan DM. Cultural cognition and synthetic biology risk perceptions: a preliminary analysis. GW Law Faculty Publications & Other Works. 2008 282. Available from: https://scholarship.law.gwu.edu/faculty_publications/282
59. Gaskell G, Stares S, Allansdottir A, Allum N, Castro P, Esmer Y, et al. Europeans and biotechnology in 2010. In: Winds of change? 2010. https://doi.org/10.2777/23393
60. Van den Belt H. Playing god in Frankenstein's footsteps: synthetic biology and the meaning of life. Nanoethics. 2009;3(3):257–68. https://doi.org/10.1007/s11569-009-0079-6 PMID: 20234875
61. Link H-J. Playing god and the intrinsic value of life: moral problems for synthetic biology? Sci Eng Ethics. 2012;19(2):435–48. https://doi.org/10.1007/s11948-012-9353-z PMID: 22389208

62. Douglas T, Powell R, Savulescu J. Is the creation of artificial life morally significant? Stud Hist Philos Sci Part C: Stud Hist Philos Biol Biomed Sci. 2013;44(4):688–96. https://doi.org/10.1016/j.shpsc.2013.05.016

63. Heavey P. Synthetic biology ethics: a deontological assessment. Bioethics. 2013;27(8):442–52. https://doi.org/10.1111/bioe.12052

64. Calvert J. The commodification of emergence: systems biology, synthetic biology and intellectual property. BioSocieties. 2008;3(4):383–98. https://doi.org/10.1017/s1745855208006303

65. Swierstra T, Rip A. Nano-ethics as NEST-ethics: patterns of moral argumentation about new and emerging science and technology. Nanoethics. 2007;1(1):3–20. https://doi.org/10.1007/s11569-007-0005-8

66. Kouper I. A critical participatory approach to the evaluation of synthetic biology. In: Ambivalences of creating life: societal and philosophical dimensions of synthetic biology. Cham: Springer International Publishing; 2015. p. 215–41. https://doi.org/10.1007/978-3-319-21088-9_11

67. Dabrock P. Playing god? Synthetic biology as a theological and ethical challenge. Syst Synth Biol. 2009;3(1-4):47–54. https://doi.org/10.1007/s11693-009-9028-5 PMID: 19816799

68. Knight AJ. Does application matter? An examination of public perception of agricultural biotechnology applications. AgBioForum. 2006;9(2):121–8. Available from: https://ssrn.com/abstract=946417

69. Gaskell G, Bard I, Allansdottir A, Da Cunha RV, Eduard P, Hampel J, et al. Public views on gene editing and its uses. Nat Biotechnol. 2017;35(11):1021–3. https://doi.org/10.1038/nbt.3958 PMID: 29121022

70. Bauer MW. Controversial medical and agri-food biotechnology: a cultivationanalysis. Public Underst Sci. 2002;11(2):93. https://dpi.org/10.1088/0963-6625/11/2/301

71. Slovic P. Trust, emotion, sex, politics, and science: Surveying the risk assessment battlefield. U. Chi. Legal F. 1997. 59 p. Available from: https://chicagounbound.uchicago.edu/uclf/vol1997/iss1/4

72. Woźniak E, Tyczewska A, Twardowski T. A shift towards biotechnology: social opinion in the EU. Trends Biotechnol. 2021;39(3):214–8. https://doi.org/10.1016/j.tibtech.2020.08.001

73. Ball P. Making life: a comment on 'Playing God in Frankenstein's footsteps: synthetic biology and the meaning of life'by Henk van den Belt (2009). Nanoethics. 2010;4(2):129–32. https://doi.org/10.1007/s11569-010-0091-x

74. Presidential Commission for the Study of Bioethical Issues. New directions. The ethics of synthetic biology and emerging technologies. Washington, DC; 2010. Available from: https://bioethicsarchive.georgetown.edu/pcsbi/sites/default/files/PCSBI-Synthetic-Biology-Report-12.16.10_0.pdf

75. Scott SE, Rozin P. Actually, natural is neutral. Nat Hum Behav. 2020;4(10):989–90. https://doi.org/10.1038/s41562-020-0891-0 PMID: 32451480

76. Rozin P, Fischler C, Shields-Argelès C. European and American perspectives on the meaning of natural. Appetite. 2012;59(2):448–55. https://doi.org/10.1016/j.appet.2012.06.001

77. Balmer A, Herreman C. Craig Venter and the re-programming of life: how metaphors shape and perform ethical discourses in the media presentation of synthetic biology. In Communicating biological sciences: ethical and metaphorical dimensions. London: Ashgate Publishing; 2009. p. 219–34.

78. Rasmussen S, Constantinescu A, Svaneborg C. Generating minimal living systems from non-living materials and increasing their evolutionary abilities. Philos Trans R Soc Lond B Biol Sci. 2016;371(1701):20150440. https://doi.org/10.1098/rstb.2015.0440 PMID: 27431518

79. Elani Y, Seddon JM. What it means to be alive: a synthetic cell perspective. Interface Focus. 2023;13(5):20230036. https://doi.org/10.1098/rsfs.2023.0036

80. Mann S. Cell mimicry: bottom-up engineering of life. Interface Focus. 2023;13(5):20230034. https://doi.org/10.1098/rsfs.2023.0034 PMID: 37577003

81. Damiano L, Stano P. On the "Life-Likeness" of synthetic cells. Front Bioeng Biotechnol. 2020;8:953. https://doi.org/10.3389/fbioe.2020.00953 PMID: 32984270

82. Schoenmakers LLJ. Scientific progress, normative discussions, and the pragmatic account of definitions of life. Synthese. 2023;201(4):139. https://doi.org/10.1007/s11229-023-04085-7

83. Friese M, Hofmann W, Schmitt M. When and why do implicit measures predict behaviour? Empirical evidence for the moderating role of opportunity, motivation, and process reliance. Eur Rev Soc Psychol. 2008;19(1):285–338. https://doi.org/10.1080/10463280802556958

84. Friese M, Wänke M, Plessner H. Implicit consumer preferences and their influence on product choice. Psychol Mark. 2006;23(9):727–40. https://doi.org/10.1002/mar.20126
